# Aqueous/Nonaqueous DBU Mixtures: Versatile Switching Media for Chemoselective Aldol, Baylis‐Hillman, and Aldol Condensation Reactions

**DOI:** 10.1002/open.202500040

**Published:** 2025-03-20

**Authors:** Elaheh Akbarzadeh, M. Saeed Abaee, Yazdanbakhsh L. Nosood, Mohammad M. Mojtahedi, Klaus Harms, Zahra Shabani

**Affiliations:** ^1^ Department of Organic Chemistry and Natural Products Chemistry and Chemical Engineering Research Center of Iran Pajouhesh Blvd., 17^th^ Km, Tehran-Karaj Highway, P.O.Box 14335–186 Tehran Iran; ^2^ Fachbereich Chemie Philipps-Universitaet Marburg Hans-Meerwein-Strasse D-35032 Marburg Germany

**Keywords:** Switching media, DBU, Chemoselective reactions, Baylis-Hillman reaction, Aldol condensation

## Abstract

Isophorone is a relatively small molecule with several neighboring reacting sites, making it susceptible to various competing reactions with aldehydes, including aldol, Baylis‐Hillman (BH), aldol condensation, and Michael addition reactions. In the present work, we have designed a switchable 1,8‐diazabicyclo[5.4.0]undec‐7‐ene (DBU)‐catalyzed procedure, where the reaction of isophorone with aldehydes is guided chemoselectively toward either aldol, BH, or aldol condensation reactions, depending on the use of water and/or heat. This controllable divergency likely stems from the ability to tune the dual nucleophilicity/basicity characters of the DBU/H_2_O medium. In other words, the nucleophilicity of DBU plays a crucial role in directing the process toward the formation of the BH adducts in the absence of water. At the same time, the aldol pathway dominates when water is present. The conditions were amenable for tandem processes, as demonstrated for an aldol condensation/Diels‐Alder sequence.

## Graphical abstract

A switchable DBU‐catalyzed procedure is designed for the reaction of isophorone with aldehydes to give either aldol, Baylis‐Hillman, or aldol condensation products, depending on the use of water and/or heat. The observed divergency stems from the ability to tune the dual nucleophilicity/basicity characters of the medium. The conditions are amenable for tandem processes, as demonstrated for an aldol condensation/Diels‐Alder sequence.

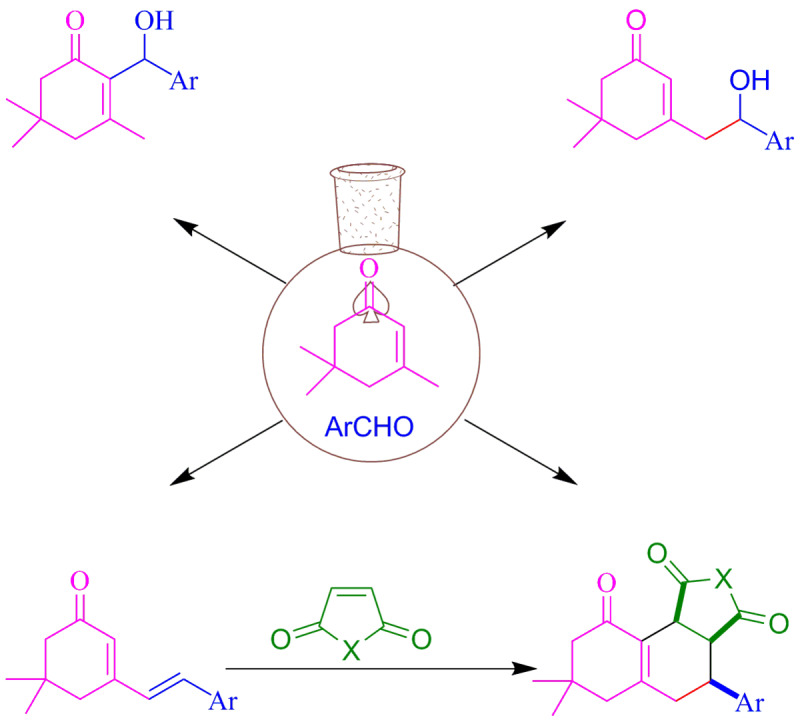

## Introduction

Organic reactions are inherently unselective due to usual presence of several active sites in the reactants with comparable enthalpies of activations.^[1]^ Additionally, prochirality and stereotopicity issues associated with the nature of specific functional groups give rise to the formation of enantiomeric and diastereomeric mixtures in many organic reactions, increasing the diversity of the products in synthetic operations.^[2]^ A recent promising, fast‐growing solution to this challenge is the application of divergent strategies in synthetic organic chemistry,^[3]^ where competent reaction pathways can be controlled so that selective and efficient formation of the desired product is achieved via the choice of conditions,^[4]^ reagents,^[5]^ catalysts,^[6]^ or by altering the order of the addition of the reactants.^[7]^ This strategy has opened a new avenue to access interesting drugs,^[8]^ molecules,^[9]^ and libraries of compounds,^[10]^ with more efficiency and convenience. On this venue, a yet promising approach to explore is taking advantage of combining sustainable chemistry with divergent synthetic strategies, such as tandem reactions,^[11]^ multicomponent procedures,^[12]^ and benign catalysis^[13]^ to get access to even more diverse structures starting from the same reactants, while waste production is minimized or even completely avoided.^[14]^

**Figure 1:**
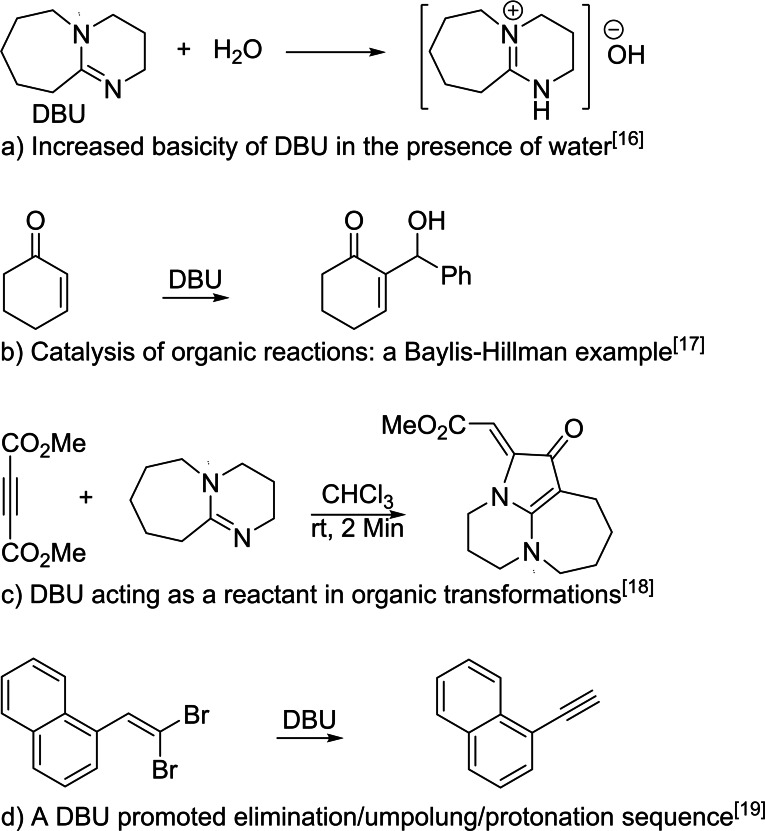
Various roles of DBU in organic synthesis.

In recent years, 1,8‐diazabicyclo[5.4.0]undec‐7‐ene (DBU) has emerged as a versatile, commercially available, inexpensive, and homogeneous reagent in synthetic chemistry to catalyze various organic reactions efficiently and under mild conditions.^[15]^ Furthermore, the performance of this reagent is not limited to its basic^[16]^ or nucleophilic catalytic^[17]^ properties, and in some cases DBU also incorporates as a reacting component in the structure of the products.^[18]^ Of particular interest is the occasional dual functioning of DBU during a single process, where it acts simultaneously as both a base and a reducing agent^[19]^ or tunes the polarity of the reaction media,^[20]^ giving rise to unexpected outcomes in some instances. Some illustrative examples are highlighted in Figure 1.

**Scheme 1:**
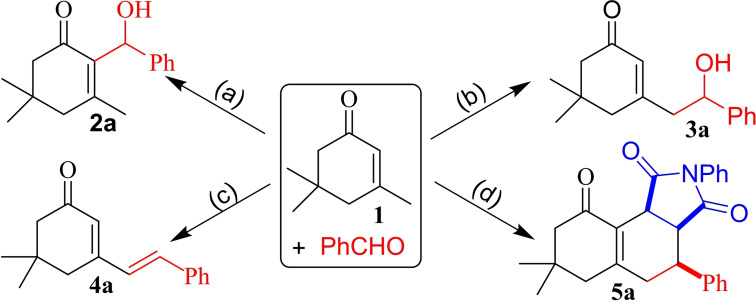
DBU/aqueous‐DBU media for diverse conversion of **1** into various products of choice. (a) DBU. (b) DBU, H_2_O. (c) DBU, H_2_O, 60 °C. (d) 1. DBU, H_2_O, 60 °C, 2. *N*‐phenylmaleimide (NPM), reflux.

Reactions of aromatic aldehydes with ketones entailing α,β‐unsaturated sites is an example of an inherently diverse process, which intrinsically produces mixtures of products in comparable yields.^[21]^ A closely related example is isophorone **1**, an α,β‐unsaturated divergent cyclic carbonyl system with several reactive functionalities, suitable as a probe to control competing parallel aldol, Baylis‐Hillman (BH), or aldol condensation reactions. In the framework of our investigations on divergent reacting systems,^22^, ^23^, ^24^ a few years ago, we introduced dihydrothiopyran‐4‐one as a related probing system using various aqueous organocatalysts.^[25]^ This background persuaded us to extend the outcome to the isophorone system. As a result of our studies, we would like to introduce a divergent procedure, where depending on the choice of the medium, the combination of **1** with aromatic aldehydes would selectively and efficiently lead to one of the several possible products (Scheme 1). Reactions take place under mild conditions, and this would obviously has a significant effect on the divergency of the process, due to the directing effects that aqueous/organocatalytic conditions would impose to switch the reaction pathways. We hereby disclose the potential of an organocatalytic system that can preferentially guide the conditions toward the desired product, depending on the presence or absence of water as a switching agent.

## Results and Discussion

Reaction of enone **1** with aldehydes gives rise to several products in comparable yields, due to the diversity in reacting sites that **1** possesses. To tackle this lack of selectivity experimentally, we evaluated the dual nucleophilicity/basicity potential of various organocatalysts as tuning agents to direct the reaction of **1** with benzaldehyde toward one of the possible pathways (Table 1). In practice, an aqueous mixture of **1** and benzaldehyde remained intact after 6 h of mixing at room temperature (entry 1). This was also the result for the solvent‐free mixture of the same reactants (entry 2). Addition of DABCO (50 mol%) to this mixture caused 35 % formation of the BH adduct **2 a** after 1 h (entry 3), while the use of Et_2_NH (entry 4), Et_3_N (entry 5), and morpholine (entry 6) was not so effective to cause the same conversion of the reactants to **2 a**. In contrast, DBU improved the outcome (entries 7–8), giving higher yields of **2 a** in the presence of lower quantities of the catalyst (entry 9). Addition of water to the same mixture of the reactants switched the direction toward the formation of aldol product **3 a** (entry 10), apparently due to the increased basicity of the DBU/H_2_O medium.^[26]^ Use of other solvents was not effective and caused either negligible (entries 11–12) or no formation of **3 a** (entries 13–14). Treatment of the latter mixture at a slightly elevated temperature continued the process to give the aldol condensation compound **4 a** (entry 15), a product that could further undergo *in situ* Diels‐Alder (DA) cycloaddition with NPM to give **5 a** (entry 16).

**Table 1:** Optimization of the conditions for various reactions of **1** with benzaldehyde.

entry	conditions	product	yield%^[a]^
1	H_2_O, rt, 6 h	none	‐
2	rt, 6 h	none	‐
3	DABCO (50 %), rt, 1 h	**2 a**	35
4	Et_2_NH (50 %), rt, 1 h	**2 a**	5
5	Et_3_N (50 %), rt, 1 h	**2 a**	5
6	morpholine (50 %), rt, 1 h	**2 a**	10
7	DBU (50 %), rt, 1 h	**2 a**	56
8	DBU (25 %), rt, 1 h	**2 a**	72
9	DBU (10 %), rt, 1 h	**2 a**	82
10	DBU (10 %), H_2_O, rt, 1 h	**3 a**	80
11	DBU (10 %), MeOH, rt, 1 h	**3 a**	<5
12	DBU (10 %), EtOH, rt, 1 h	**3 a**	<5
13	DBU (10 %), THF, rt, 1 h	**3 a**	‐
14	DBU (10 %), PhMe, rt, 1 h	**3 a**	‐
15	DBU (10 %), H_2_O, 60 °C, 2 h	**4 a**	82
16	DBU (10 %), H_2_O, 60 °C; NPM, reflux, 24 h	**5 a**	80

[a] Isolated yields.

Having the optimized conditions for each pathway in hand, we next examined the synthetic scope of each reaction separately. In other words, reactions of **1** with aldehydes in the absence of water would produce BH adducts, while addition of H_2_O to the same mixture of the reactants leads to aldol reaction products (Table 2). Thus, under nonaqueous conditions, benzaldehyde (entry 1) and other aromatic aldehydes bearing electron‐releasing (entries 2–4) or electron‐withdrawing (entries 5–6) groups would react with **1** to give the respective products **2 a**‐**f** in 80–88 % yields and within 1 h of mixing at rt. In contrast, addition of water to the same mixtures of the reactants would produce high yields of **3 a**‐**f** (entries 7–12).

**Table 2:** Switching BH/aldol reactions of **1** with aromatic aldehydes under catalytic DBU or DBU/H_2_O conditions.

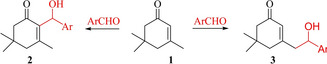			
entry	Ar	product	yield%^[a]^
1	C_6_H_5_	**2 a** ^[b]^	82
2	3‐MeC_6_H_4_	**2 b** ^[b]^	84
3	4‐MeC_6_H_4_	**2 c** ^[b]^	82
4	4‐MeOC_6_H_4_	**2 d** ^[b]^	80
5	3‐MeOC_6_H_4_	**2 e** ^[b]^	86
6	2‐FC_6_H_4_	**2 f** ^[b]^	88
7	C_6_H_5_	**3 a** ^[c]^	80
8	3‐MeC_6_H_4_	**3 b** ^[c]^	79
9	4‐MeC_6_H_4_	**3 c** ^[c]^	86
10	4‐MeOC_6_H_4_	**3 d** ^[c]^	82
11	3‐MeOC_6_H_4_	**3 e** ^[c]^	85
12	2‐FC_6_H_4_	**3 f** ^[c]^	84

[a] Isolated yields. [b] DBU, rt, 1 h. [c] DBU/H_2_O, rt, 1 h.

**Figure 2:**
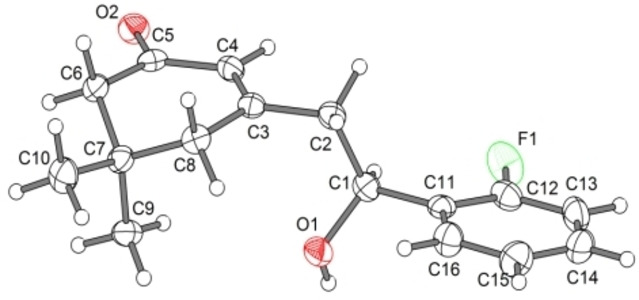
X‐ray crystal structure of product **3 f**. Thermal ellipsoids set at 50 % probability level. Disordered positions are not shown.

The structures of the two sets of structural isomeric products were elucidated based on their ^1^H‐NMR and ^13^C‐NMR spectra. For example, formation of a benzylic CH and the adjacent OH moiety was apparent in the ^1^H‐NMR spectra of both series. In addition, the three Me groups in the starting ketone still appeared in the spectra of the products in the case of **2**, while the allylic Me disappeared in the spectra of the products **3**, due to conversion to CH_2_ moieties. The structure of **3** was further confirmed by X‐ray crystallography analysis of the single crystals of **3 f** (Figure 2).

**Figure 3:**
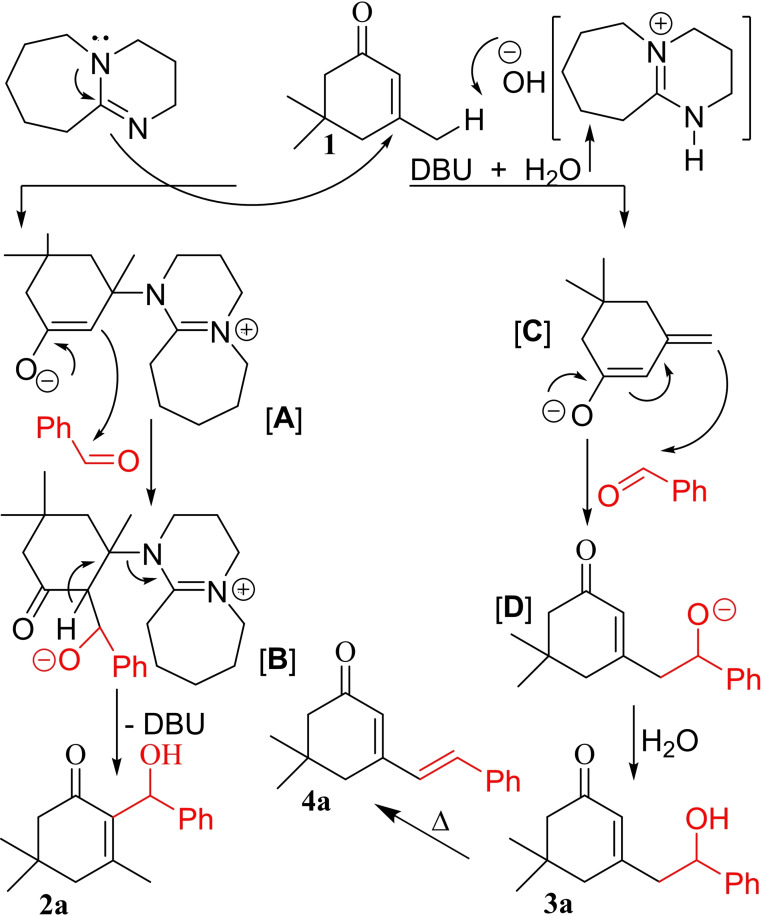
A plausible mechanism for the formation of **2** and **3**.

Based on our observations and according to literature reports regarding the distinct behaviors of DBU under aqueous^[27]^ or nonaqueous conditions,^[28]^ a mechanism would be proposed for the observed chemoselectivity under dry or H_2_O conditions, as shown in Figure 3 for the reaction of **1** with benzaldehyde. Under dry conditions, DBU, as a nucleophile, directly attacks the olefinic β‐position site of **1** to give the intermediate **A**. Then, enolate **A** adds to the aldehyde to give **B**, which upon facile departure of DBU and O‐protonation gives the BH adducts **2 a** (left pathway). In contrast, the relatively strong basic hydrated DBU moiety is formed when water is present in the mixture. This species is sterically hindered and preferably acts as a base to remove the remote acidic proton to give the dienolate intermediate **C**. In continuation, **C** attacks the aldehyde to complete the aldol pathway, giving either **3 a** or **4 a**, depending on the temperature of the reaction mixture (right pathway). Regarding the last step, catalysis of DA reactions with amines is well known and documented in the literature.^[29]^

To further illustrate the divergency of this approach, we then continued the aldol pathway to get the final condensation products by slightly increasing the reaction temperature. For this purpose, the aqueous mixtures of the reactants were heated to 60 ^°C^, resulting in convenient formation of the dienone products **4 a**‐**h** within 2 h reaction periods (Table 3).

**Table 3:** DBU/H_2_O catalyzed aldol condensation reactions of **1**.

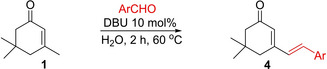
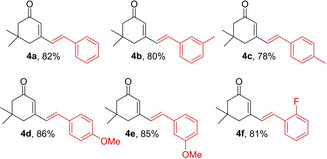

Finally, upon addition of a dienophile to the latter reaction mixtures and continuing the process at refluxing temperature, the respective DA adducts were formed in good yields. For this part, reactions of **1** with three model aldehydes (C_6_H_5_CHO, 4‐MeC_6_H_4_CHO, and 2‐thienylCHO) were carried out and the dienophiles (NPM, maleic anhydride, methyl acrylate, and dimethyl acetylenedicarboxylate) were added to the mixtures, after TLC showed initial formation of the respective diene intermediates. Products **5–8** were formed in high yields after 12 to 36 h of refluxing in DBU/H_2_O mixtures (Table 4).

**Table 4:**
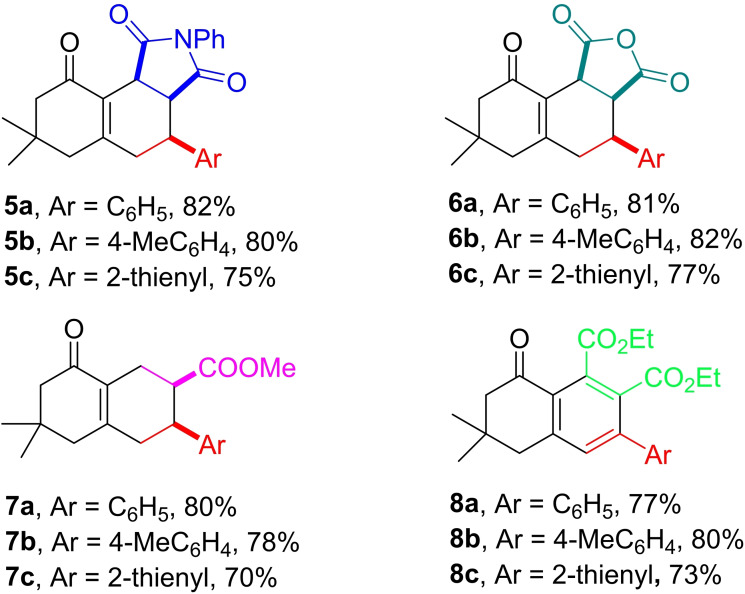
One‐pot aldol condensation‐DA sequence starting from **1** to get **5–8**.

## Conclusions

In summary, we demonstrated that aqueous/nonaqueous DBU catalysis would be an efficient solution to guide reactions of unselective systems toward one of the possible pathways so that a product that is usually formed in low yield under conventional procedures is chemoselectively obtained using the DBU or DBU/H_2_O conditions. In other words, the differential reactivity of DBU in nonaqueous mixtures, as compared to aqueous conditions, prevents the formation of mixtures of products using the present protocol. While dry DBU is capable to direct the combination of isophorone and the aldehyde toward BH addition, hydrated DBU prefers to cause aldol reaction between the same two reactants. In addition, continuation of the aqueous reactions at elevated temperatures opens the pathway for the formation of the respective dienes and even the following tandem DA cycloadditions. Conditions are mild, the procedure is easy and fast, and the reagent is commercially available.

## Experimental Section

### General

All ^1^H‐NMR and ^13^C‐NMR spectra are recorded using FT‐NMR Bruker Avance (300 MHz) spectrometer in CDCl_3_ solutions. The chemical shifts are expressed as δ units with tetramethylsilane (TMS) as the internal standard (δ=0). Chemical shifts are reported in parts per million (δ, ppm). Multiplicities are expressed as s (singlet), d (doublet), t (triplet), dd (double doublet), and so on. Fourier transform infrared (FTIR) spectra are recorded in potassium bromide (KBr) pellets on a Perkin Elmer FT spectrum RX1 (Perkin Elmer, Norwalk, CT, USA) and Bruker (IFS‐88) over the range of 400–4000 cm^−1^. Mass spectra are obtained on a Finnigan Mat 8430 apparatus at an ionization potential of 70 eV. Elemental analysis was performed by a Perkin Elmer 2004 (II) and ThermoFinnigan Flash EA 1112 CHN analyzer. Melting points (uncorrected) are measured with an Electrothermal Engineering LTD 9200 apparatus (Essex, UK). Chemicals are purchased from Merck and used without further purification. Aldehydes are purified prior to use, using standard methods. Column chromatography experiments are carried out on silica gel 60 (0.06‐0.2 mm) using EtOAc/hexanes solutions. New products were fully characterized. Known products (**2 a**, **3 a**, **3 c**‐**d**, **4–8**) are known and were partially characterized and identified by the comparison of their data with those of authentic samples.^21^, ^30^

### Typical Procedure for the BH Reactions

A mixture of **1** (300 μL, 2.0 mmol), benzaldehyde (284 μL, 2.0 mmol), and DBU (15 μL, 10 mol%) was stirred at room temperature for 1 h. After completion of the reaction (monitored by TLC using EtOAc/hexanes (1 : 4) as the eluent), the mixture was extracted with EtOAc (3×5 mL), washed with brine, dried over anhydrous Na_2_SO_4_, and concentrated under reduced pressure. The residue was fractionated to obtain **2 a** (400 mg) using column chromatography (EtOAc/hexanes, 1 : 4, v/v).

### Typical Procedure for the Aldol Reactions

A mixture of **1** (300 μL, 2.0 mmol), benzaldehyde (284 μL, 2.0 mmol), water (1 mL), and DBU (15 μL, 10 mol%) was stirred at room temperature for 1 h. After completion of the reaction (monitored by TLC using EtOAc/hexanes (1 : 4) as the eluent), the mixture was extracted with EtOAc (3×5 mL), washed with brine, dried over anhydrous Na_2_SO_4_, and concentrated under reduced pressure. The residue was fractionated to obtain **3 a** (398 mg) using column chromatography (EtOAc/hexanes, 1 : 4, v/v).

### Typical Procedure for the Aldol Condensations

A mixture of **1** (300 μL, 2.0 mmol), benzaldehyde (284 μL, 2.0 mmol), water (1 mL), and DBU (15 μL, 10 mol%) was stirred at 60 °C for 2 h. After completion of the reaction (monitored by TLC using EtOAc/hexanes (1 : 4) as the eluent), the mixture was extracted with EtOAc (3×5 mL), washed with brine, dried over anhydrous Na_2_SO_4_, and concentrated under reduced pressure. The residue was fractionated to obtain **4 a** (371 mg) using column chromatography (EtOAc/hexanes, 1 : 4, v/v).

### Typical Procedure for DA Reactions

A mixture of **1** (300 μL, 2.0 mmol), benzaldehyde (284 μL, 2.0 mmol), water (1 mL), and DBU (15 μL, 10 mol%) was stirred at 60 °C for 2 h. A dienophile (3.0 mmol of NPM, MA, MAC, or DMAD) was added to the reaction vessel and the resulting mixture was refluxed for appropriate length of time (monitored by TLC using EtOAc/hexanes (1 : 4) as the eluent). The mixture was extracted with EtOAc (3×5 mL), washed with brine, dried over anhydrous Na_2_SO_4_, and concentrated under reduced pressure. The residue was fractionated to obtain the respective DA adduct (**5 a**‐**8 a**) using column chromatography (EtOAc/hexanes, 1 : 4, v/v).

### Spectral Data of New Products

#### 2‐(Hydroxy(m‐tolyl)methyl)‐3,5,5‐trimethylcyclohex‐2‐enone (2 b)

Liquid, 433 mg, 84 %; IR (ν_max_ KBr, cm^−1^): 1652, 2921, 2957, 3434; ^1^H‐NMR (300 MHz, CDCl_3_): δ=1.03 (s, 3H), 1.07 (s, 3H), 2.04 (s, 3H), 2.27 (s, 2H), 2.34 (s, 3H), 2.35 (s, 2H), 4.82 (d, *J*=10.5 Hz, 1H), 5.65 (d, *J*=10.5 Hz, 1H), 7.05 (d, *J*=7.5 Hz, 1H), 7.07 (d, *J*=7.5 Hz, 1H), 7.19 (s, 1H), 7.21 (dd, *J*=7.5, 7.5 Hz, 1H) ppm; ^13^C‐NMR (75 MHz, CDCl_3_): δ=21.2, 21.5, 28.0, 28.2, 32.8, 47.2, 51.8, 71.1, 122.2, 126.0, 127.6, 128.1, 134.1, 137.8, 143.4, 155.9, 201.7 ppm; MS (70 eV) m/z (%), 258 (M^+^), 77, 91, 139, 157, 198, 242; Elemental analysis: Calculated for C_17_H_22_O_2_; C, 79.03; H, 8.58; found C, 79.15; H, 8.86.

#### 2‐(Hydroxy(p‐tolyl)methyl)‐3,5,5‐trimethylcyclohex‐2‐enone (2 c)

Liquid, 423 mg, 82 %; IR (ν_max_ KBr, cm^−1^): 1651, 2868, 2956, 345; ^1^H‐NMR (300 MHz, CDCl_3_): δ=1.02 (s, 3H), 1.07 (s, 3H), 2.03 (s, 3H), 2.26 (d, *J*=14.5 Hz, 1H), 2.27 (d, *J*=14.5 Hz, 1H), 2.31 (s, 3H), 2.34 (s, 2H), 4.83 (d, *J*=10.5 Hz, 1H), 5.65 (d, *J*=10.5 Hz, 1H), 7.12 (d, *J*=8.0 Hz, 2H), 7.22 (d, *J*=8.0 Hz, 2H) ppm; ^13^C‐NMR (75 MHz, CDCl_3_): δ=21.0, 21.2, 28.1, 28.2, 32.8, 47.3, 51.9, 71.0, 125.2, 128.9, 134.1, 136.4, 140.5, 155.8, 201.6 ppm; MS (70 eV) m/z (%), 258 (M^+^), 77, 91, 118, 140, 150, 164, 196, 242; Elemental analysis: Calculated for C_17_H_22_O_2_; C, 79.03; H, 8.58; found C, 79.23; H, 8.90.

#### 2‐(Hydroxy(4‐methoxyphenyl)methyl)‐3,5,5‐trimethylcyclohex‐2‐enone (2 d)

Liquid; 449 mg, 82 %; IR (ν_max_ KBr, cm^−1^): 1651, 2867, 2957, 3437; ^1^H‐NMR (300 MHz, CDCl_3_): δ=1.01 (s, 3H), 1.05 (s, 3H), 2.00 (s, 3H), 2.25 (s, 2H), 2.32 (s, 2H), 3.76 (s, 3H), 4.86 (br s, 1H), 5.62‐5.64 (m, 1H), 6.83 (d, *J*=8.5 Hz, 2H), 7.24 (d, *J*=8.5 Hz, 2H) ppm; ^13^C‐NMR (75 MHz, CDCl_3_): δ=21.1, 28.0, 28.1, 32.8, 47.2, 51.8, 55.1, 70.8, 113.5, 126.5, 134.0, 135.6, 155.8, 158.4, 201.7 ppm; MS (70 eV) m/z (%), 274 (M^+^), 77, 108, 134, 150, 171, 198, 258; Elemental analysis: Calculated for C_17_H_22_O_3_; C, 74.42; H, 8.08; found C, 74.19; H, 7.89.

#### 2‐(Hydroxy(3‐methoxyphenyl)methyl)‐3,5,5‐trimethylcyclohex‐2‐enone (2 e)

Liquid; 471 mg, 86 %; IR (ν_max_ KBr, cm^−1^): 1650, 1654, 3435; ^1^H‐NMR (300 MHz, CDCl_3_): δ=1.03 (s, 3H), 1.06 (s, 3H), 2.04 (s, 3H), 2.26 (d, *J*=14.5 Hz, 1H), 2.28 (d, *J*=14.5 Hz, 1H), 2.35 (s, 2H), 3.80 (s, 3H), 4.79 (d, *J*=10.0 Hz, 1H), 7.65 (d, *J*=10.0 Hz, 1H), 6.76 (dd, *J*=2.0, 8.0 Hz, 1H), 6.87 (d, *J*=8.0 Hz, 1H), 6.94 (d, *J*=2.0 Hz,1H), 7.21 (dd, *J*=8.0, 8.0 Hz, 1H) ppm; ^13^C‐NMR (75 MHz, CDCl_3_): δ=21.2, 28.0, 28.3, 32.8, 47.2, 51.8, 55.1, 70.9, 111.1, 112.2, 117.5, 129.2, 134.0, 145.2, 156.1, 159.6, 201.6 ppm; MS (70 eV) m/z (%), 274 (M^+^), 109, 139, 258; Elemental analysis: Calculated for C_17_H_22_O_3_; C, 74.42; H, 8.08; found C, 74.54; H, 8.31.

#### 2‐((2‐Fluorophenyl)(hydroxy)methyl)‐3,5,5‐trimethylcyclohex‐2‐enone (2 f)

Liquid; 461 mg, 88 %; IR (ν_max_ KBr, cm^−1^): 1650, 1726, 3448; ^1^H‐NMR (300 MHz, CDCl_3_): δ=0.95 (s, 3H), 1.04 (s, 3H), 2.07 (s, 3H), 2.23 (s, 2H), 2.29 (s, 1H), 2.31 (s, 1H), 5.05 (d, *J*=10.5 Hz, 1H), 5.92 (d, *J*=10.5 Hz, 1H), 6.94 (ddd, *J*=1.5, 7.5, 11.0 Hz, 1H), 7.14 (ddd, *J*=1.5, 7.5, 8.5 Hz, 1H), 7.20‐7.24 (m, 1H), 7.60 (ddd, *J*=1.5, 7.5, 8.0 Hz, 1H) ppm; ^13^C‐NMR (75 MHz, CDCl_3_): δ=21.2, 27.8, 28.2, 32.8, 47.4, 51.9, 66.4 (d, *J*=2.5 Hz), 115.0 (d, *J*=22.0 Hz), 124.0 (d, *J*=3.5 Hz), 128.0 (d, *J*=3.5 Hz), 128.5, 128.6, 130.1, 132.9, 159.2 (d, *J*=255.0 Hz), 202.0 ppm; MS (70 eV) m/z (%), 262 (M^+^), 109, 246; Elemental analysis: Calculated for C_16_H_19_FO_2_; C, 73.26; H, 7.30; found C, 73.41; H, 7.20.

#### 3‐(2‐Hydroxy‐2‐m‐tolylethyl)‐5,5‐dimethylcyclohex‐2‐enone (3 b)

Liquid; 408 mg, 79 %; IR (ν_max_ KBr, cm^−1^): 3396, 2955, 1653; ^1^H‐NMR (300 MHz, CDCl_3_): δ=1.00 (s, 6H), 2.19 (s, 2H), 2.23 (s, 2H), 2.33 (s, 3H), 2.35 (s, 1H), 2.53 (dd, *J*=4.5, 14.0 Hz, 1H), 2.65 (dd, *J*=8.5, 14.0 Hz, 1H), 4.86 (dd, *J*=4.5, 8.5 Hz, 1H), 5.93 (s, 1H), 7.10 (d, *J*=7.5 Hz, 1H), 7.12‐7.15 (m, 2H), 7.24 (dd, *J*=7.5, 7.5 Hz, 1H) ppm; ^13^C‐NMR (75 MHz, CDCl_3_): δ=21.3, 28.1, 28.2, 33.5, 44.2, 47.4, 50.8, 72.4, 122.7, 126.3, 126.7, 128.5, 128.6, 138.2, 143.5, 160.3, 200.0 ppm; MS (70 eV) m/z (%), 258 (M^+^), 242, 140; Elemental analysis: Calculated for C_17_H_22_O_2_; C, 79.03; H, 8.58; found C, 79.18; H, 8.37.

#### 3‐(2‐Hydroxy‐2‐(3‐methoxyphenyl)ethyl)‐5,5‐dimethylcyclohex‐2‐enone (3 e)

Liquid; 466 mg, 85 %; IR (ν_max_ KBr, cm^−1^): 3400, 1659, 1601; ^1^H‐NMR (300 MHz, CDCl_3_): δ=0.97 (s, 6H), 2.14 (s, 2H), 2.20 (s, 2H), 2.50 (dd, *J*=4.5, 14.0 Hz, 1H), 2.61 (dd, *J*=8.5, 14.0 Hz, 1H), 2.93 (br s, 1H), 3.77 (s, 3H), 4.83 (dd, *J*=4.5, 8.5 Hz, 1H), 5.89 (s, 1H), 6.79 (dd, *J*=1.5, 8.0 Hz, 1H), 6.88‐6.90 (m, 2H), 7.23 (dd, *J*=8.0, 8.0 Hz, 1H) ppm; ^13^C‐NMR (75 MHz, CDCl_3_): δ=28.0, 28.2, 33.4, 44.1, 47.4, 50.7, 55.1, 72.1, 111.2, 113.1, 117.9, 126.5, 129.5, 145.4, 159.6, 160.5, 200.1 ppm; MS (70 eV) m/z (%), 274 (M^+^), 257, 140, 123, 109, 77; Elemental analysis: Calculated for C_17_H_22_O_3_; C, 74.42; H, 8.08; found C, 74.21; H, 8.30.

#### 3‐(2‐(2‐Fluorophenyl)‐2‐hydroxyethyl)‐5,5‐dimethylcyclohex‐2‐enone (3 f)

White crystals, mp: 86–88 °C; 440 mg, 84 %; IR (ν_max_ KBr, cm^−1^): 3344, 2961, 2940, 1637, 1587; ^1^H‐NMR (300 MHz, CDCl_3_): δ=0.98 (s, 3H), 0.99 (s, 3H), 2.16 (s, 2H), 2.24 (s, 2H), 2.59 (s, 1H), ), 2.61 (s, *1*H), 2.95 (s, 1H), 5.21 (br s, 1H), 5.89 (s, 1H), 6.99 (dd, *J*=7.5, 7.5 Hz, 1H), 7.14 (d, *J*=7.5 Hz, 1H), 7.20‐7.27 (m, 1H), 7.48 (dd, *J*=7.5, 7.5 Hz, 1H) ppm; ^13^C‐NMR (75 MHz, CDCl_3_): δ=28.1, 28.2, 33.5, 44.0, 46.4, 50.8, 65.9 (d, *J*=2.5 Hz), 115.2 (d, *J*=21.5), 124.4 (d, *J*=3.5), 126.8, 127.0 (d, *J*=4.5), 129.1 (d, *J*=8.0), 130.7 (d, *J*=13.0), 158.5 (d, *J*=245.0), 160.3, 200.3 ppm; MS (70 eV) m/z (%), 262 (M^+^), 139, 123, 110, 77; Anal Calcd for C_16_H_19_FO_2_: C, 73.26; H, 7.30. Found: C, 72.97; H, 7.11; Elemental analysis: Calculated for C_16_H_19_FO_2_; C, 73.26; H, 7.30; found C, 73.15; H, 7.42.

### Single Crystal X‐ray Structure Determinations of Compounds 3 f

Crystal structure of **3 f**. Single crystals of **3 f** suitable for SC‐XRD measurement were grown by slow evaporation of an EtOAc solution of the compound. Data was collected with a Bruker D8 QUEST diffractometer equipped with MoK_α_ radiation, a graded multilayer mirror monochromator (λ = 0.71073 Å), and a Photon 100 area detector using an oil‐coated shock‐cooled crystal at 100(2) K; crystal dimensions: 0.66×0.58×0.38 mm. The unit cell dimensions were determined from 4380 reflections. The structure was solved by direct method and refined by full matrix least‐squares calculations based on F^2^ to final R1=0.037 (observed data) and wR2 (all data)=0.083, using SHELXS‐97 and SHELXL‐2012. The compound is crystallized at orthorhombic system and Pca2_1_ space group. One independent molecule with molecular formula of C_16_H_19_FO_2_ was found in the asymmetric unit, giving a total Z=4 for the unit cell; a=12.6816(10), b=6.3773(5), c=16.8780(14), cell volume V=1365.00(19)Å3. The non‐hydrogen atoms have been refined anisotropically, carbon bonded hydrogen atoms were included at calculated positions and refined using the ‘riding model’ with isotropic temperature factors at 1.2 times (for CH_3_ groups 1.5 times) that of the preceding carbon atom. CH_3_ groups were allowed to rotate about the bond to their next atom to fit the electron density. Disorder has been refined for the hydroxyl group and the position of the fluorine atom (ratio 0.85:0.15). The oxygen bonded hydrogen atom of the main part was located and allowed to refine isotropically. The OH group and the F atom of the minor disordered part were idealized using the geometry and temperature factors of the main part. Deposition Number2403157 (for **3 f**) contains the supplementary crystallographic data for this paper. These data are provided free of charge by the joint Cambridge Crystallographic Data Centre and Fachinformationszentrum Karlsruhe Access Structures service.

## Conflict of Interests

The authors declare no conflict of interest.

## Supporting information

As a service to our authors and readers, this journal provides supporting information supplied by the authors. Such materials are peer reviewed and may be re‐organized for online delivery, but are not copy‐edited or typeset. Technical support issues arising from supporting information (other than missing files) should be addressed to the authors.

Supporting Information

## Data Availability

The data that support the findings of this study are available in the supplementary material of this article.
