# Supplementary material for: Aqueous/Nonaqueous DBU Mixtures: Versatile Switching Media for Chemoselective Aldol, Baylis‐Hillman, and Aldol Condensation Reactions
Source: ChemistryOpen. 2025 Mar 20;14(9):e202500040. doi: 10.1002/open.202500040 (PMC12820351; doi:10.1002/open.202500040)

ChemistryOpen

Supporting Information

Aqueous/Nonaqueous DBU Mixtures: Versatile Switching Media for Chemoselective Aldol, Baylis-Hillman, and Aldol Condensation Reactions

Elaheh Akbarzadeh, M. Saeed Abaee,* Yazdanbakhsh L. Nosood, Mohammad M. Mojtahedi, Klaus Harms, and Zahra Shabani

Supporting Information

Aqueous/Nonaqueous DBU Mixtures: Versatile Switching Media for Chemoselective Aldol, Baylis-Hillman, and Aldol Condensation Reactions

Elaheh Akbarzadeh,[a] M. Saeed Abaee,[a] Yazdanbakhsh L. Nosood,[a] Mohammad M. Mojtahedi,[a] Klaus Harms,[b] and Zahra Shabani[a]

[a]Department of Organic Chemistry and Natural Products, Chemistry and Chemical Engineering Research Center of Iran
Pajouhesh Blvd., 17th Km, Tehran-Karaj Highway, P.O.Box 14335-186, Tehran, Iran, 1496813151
E-mail: abaee@ccerci.ac.ir
[b]Fachbereich Chemie , Philipps-Universitaet Marburg, Hans-Meerwein-Strasse, D-35032 Marburg, Germany

Spectra for New Compounds

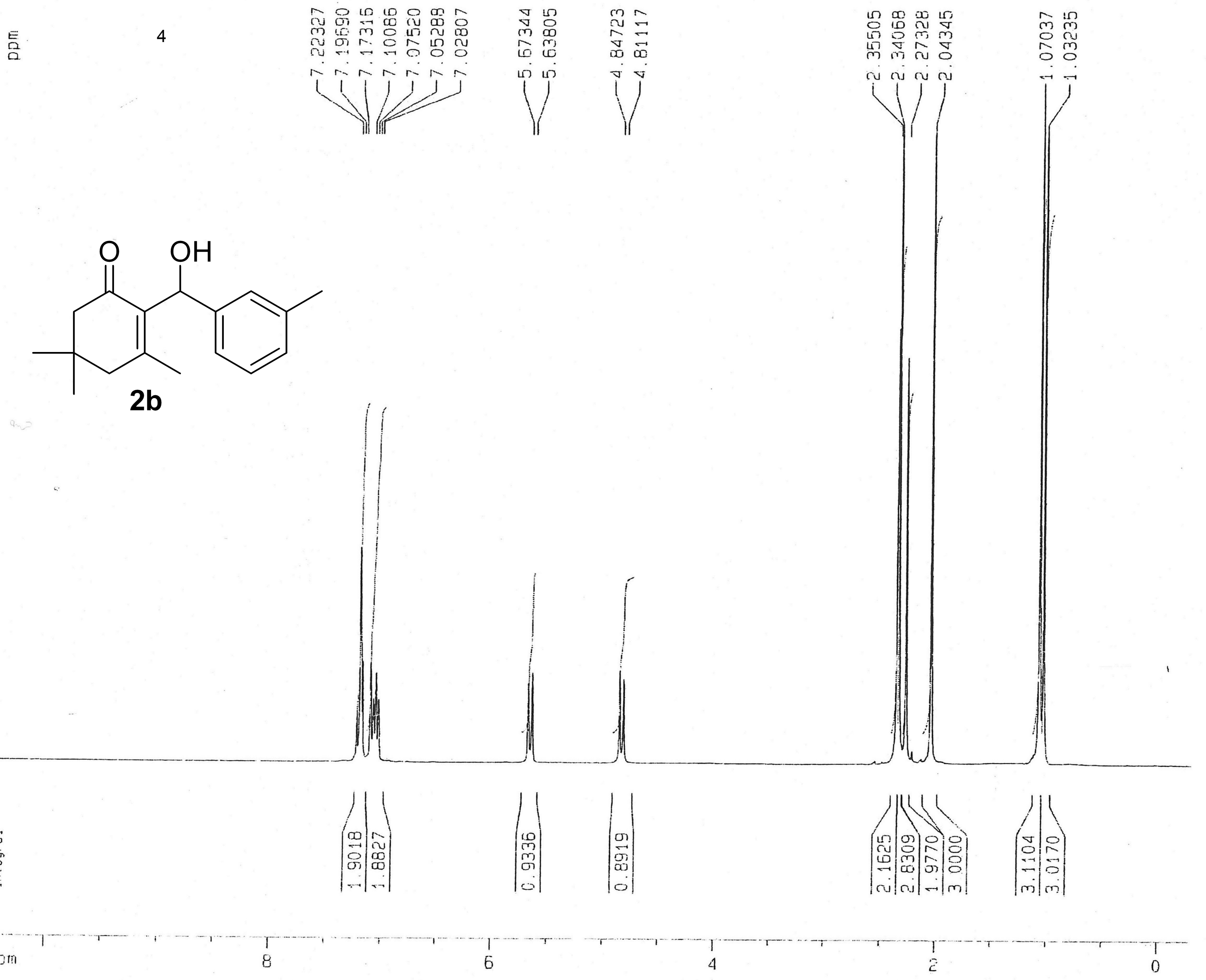
ppm
4
7.22327
7.19690
7.17315
7.10086
7.07520
7.05288
7.02807
5.67344
5.63805
4.84723
4.81117
2.35505
2.34068
2.27328
2.04345
1.07037
1.03235
O
OH
2b
1.9018
1.8827
0.9336
0.8919
2.1625
2.8309
1.9770
3.0000
3.1104
3.0170
8
6
4
2
0

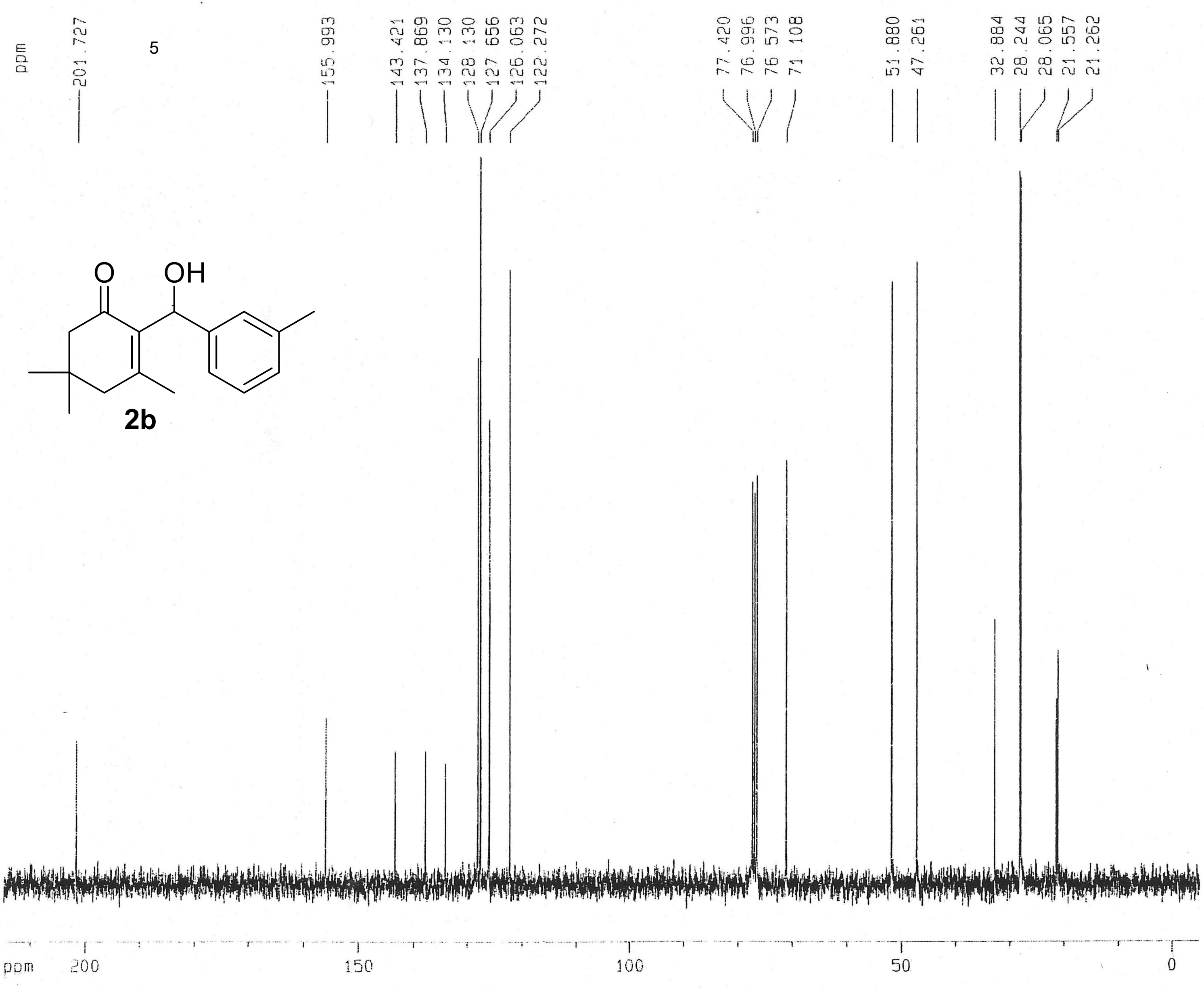
ppm
201.727
5
155.993
143.421
137.869
134.130
128.130
127.656
126.063
122.272
77.420
76.996
76.573
71.108
51.880
47.261
32.884
28.244
28.065
21.557
21.262
O
OH
2b
ppm
200
150
100
50
0

6

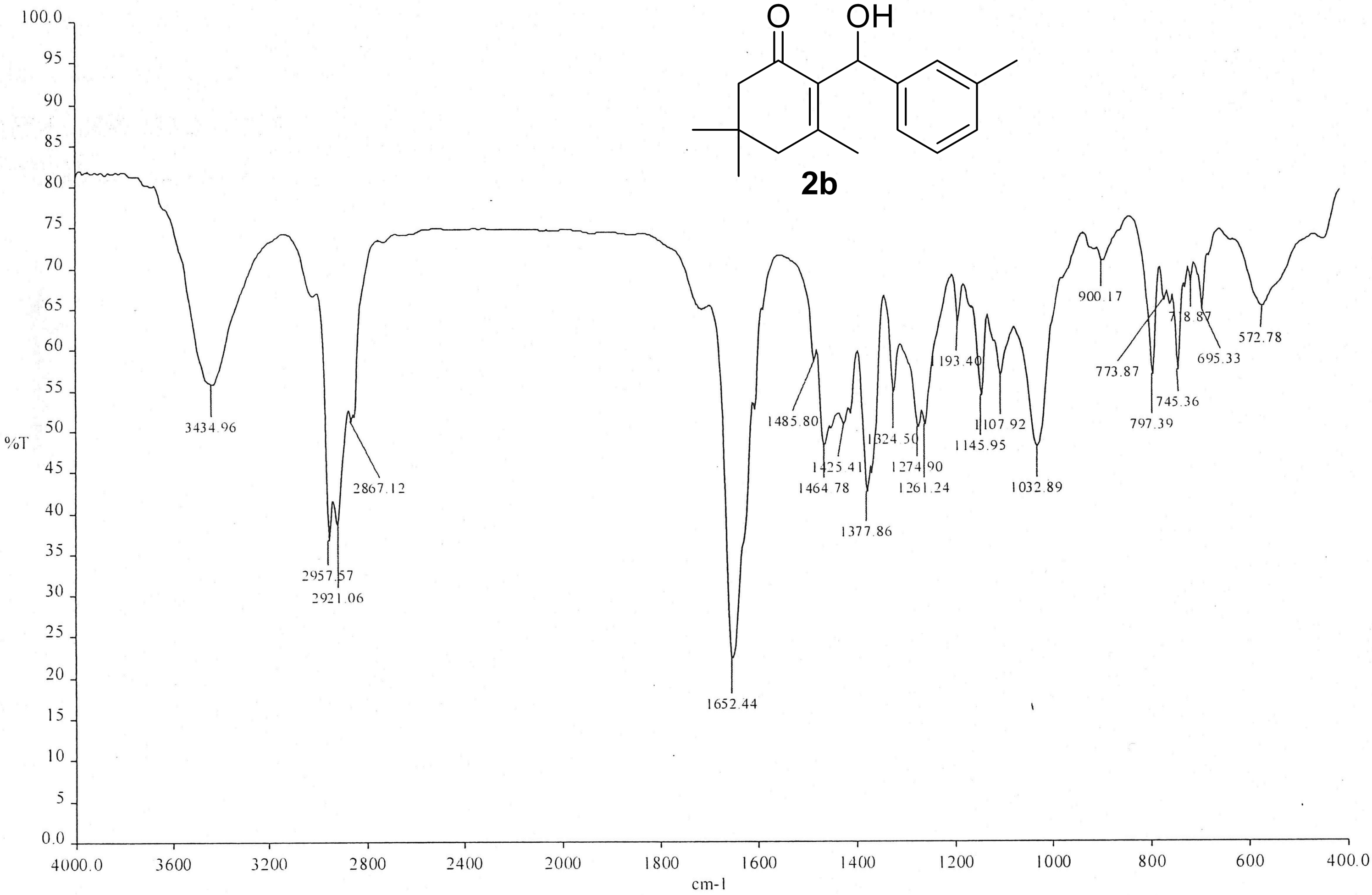

Ms. Akbarzade Sample: bis 34(bh)

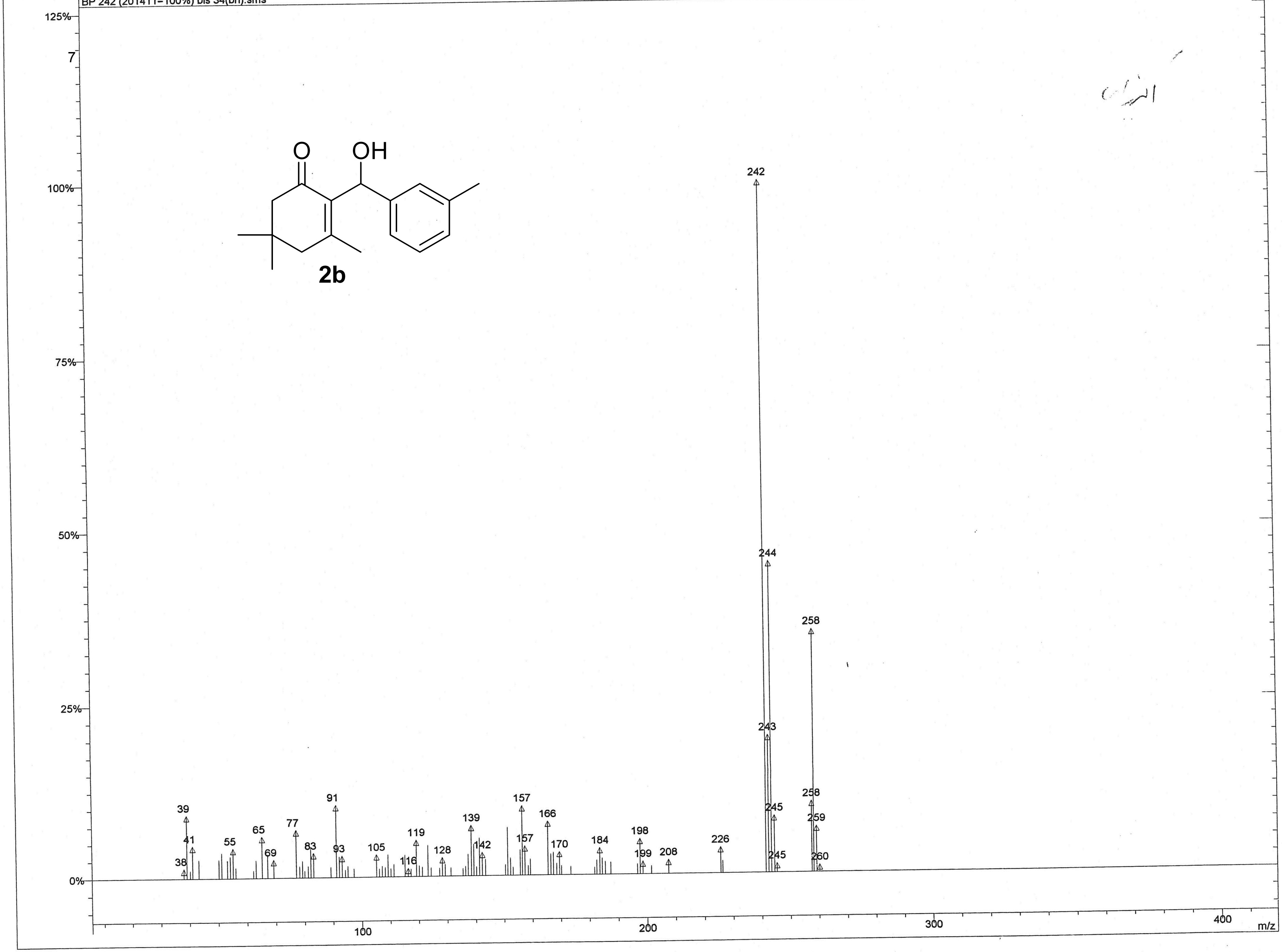
125%
100%
75%
50%
25%
0%
OH
O
2b
242
244
258
243
258
245
259
245
260
39
41
38
55
65
69
77
83
91
93
105
116
119
128
139
142
157
157
166
170
184
198
199
208
226
100
200
300
400
m/z

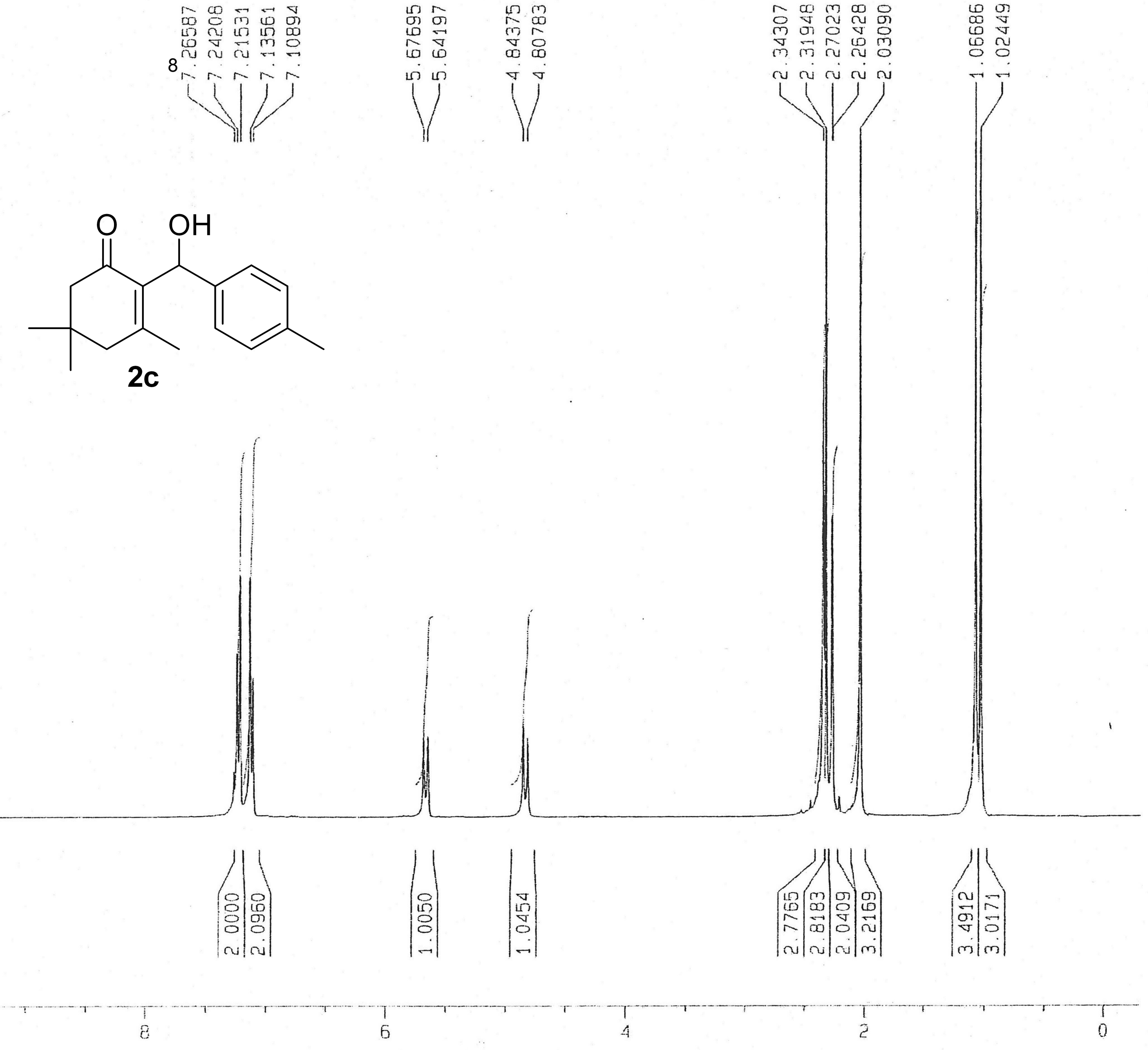

8
7.26587
7.24208
7.21531
7.13561
7.10894
5.67695
5.64197
4.84375
4.80783
2.34307
2.31948
2.27023
2.26428
2.03090
1.06686
1.02449
O
OH
2c
2.0000
2.0960
1.0050
1.0454
2.7765
2.8183
2.0409
3.2169
3.4912
3.0171
8
6
4
2
0

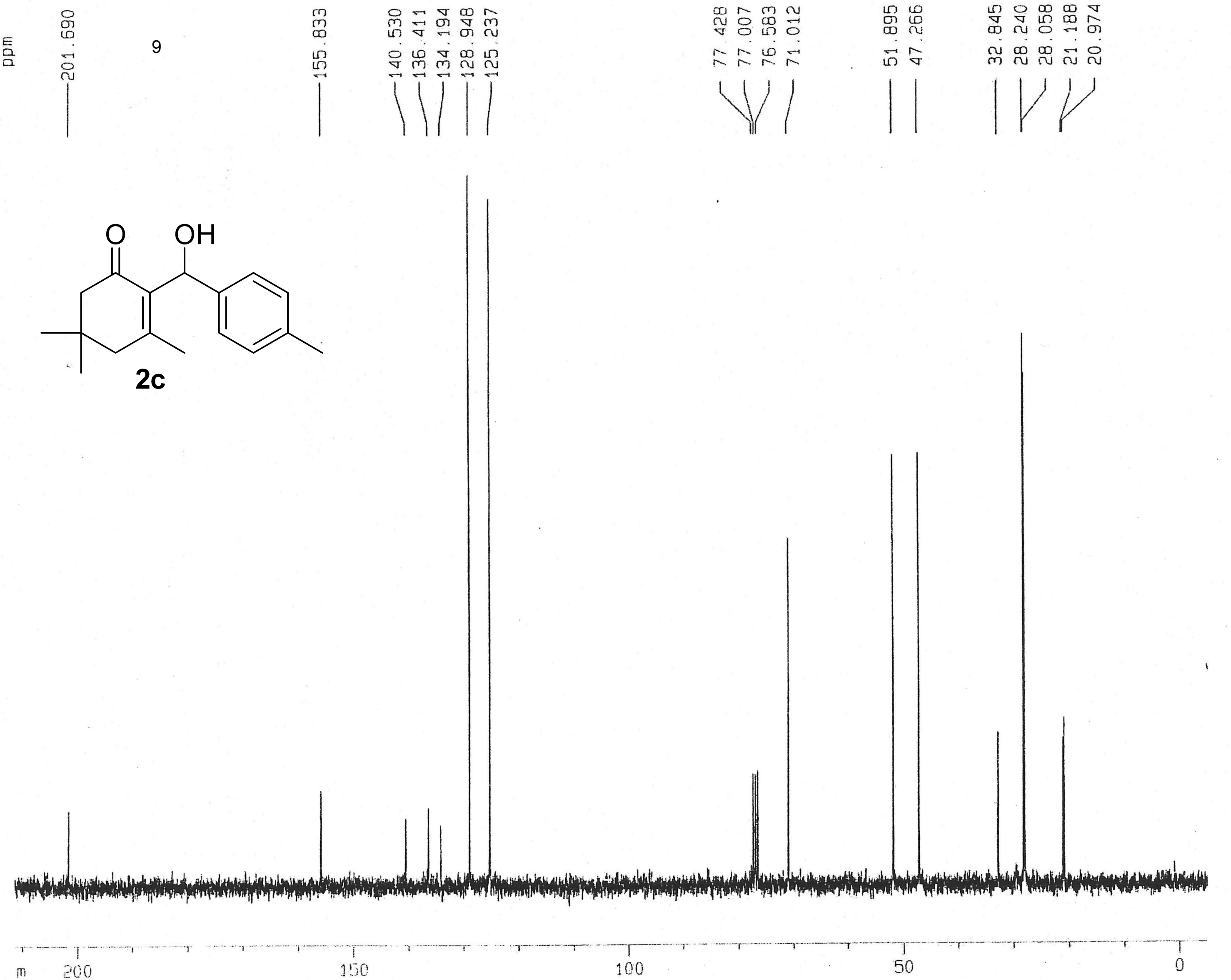
ppm
201.690
155.833
140.530
136.411
134.194
128.948
125.237
77.428
77.007
76.583
71.012
51.895
47.266
32.845
28.240
28.058
21.188
20.974
O
OH
200
150
100
50
0

2c

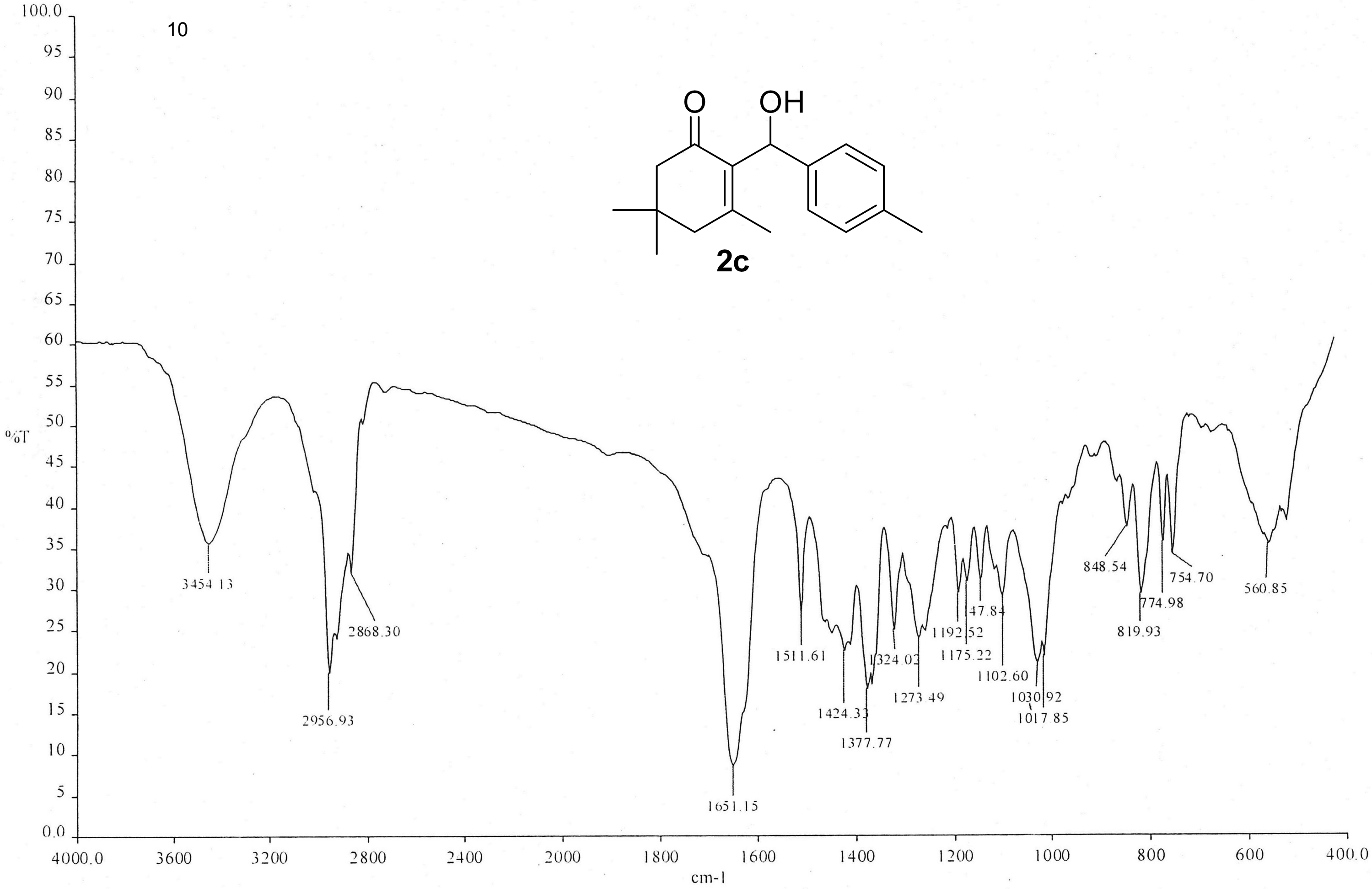

Ms. Akbarzadeh Sample: bh(4-Me)

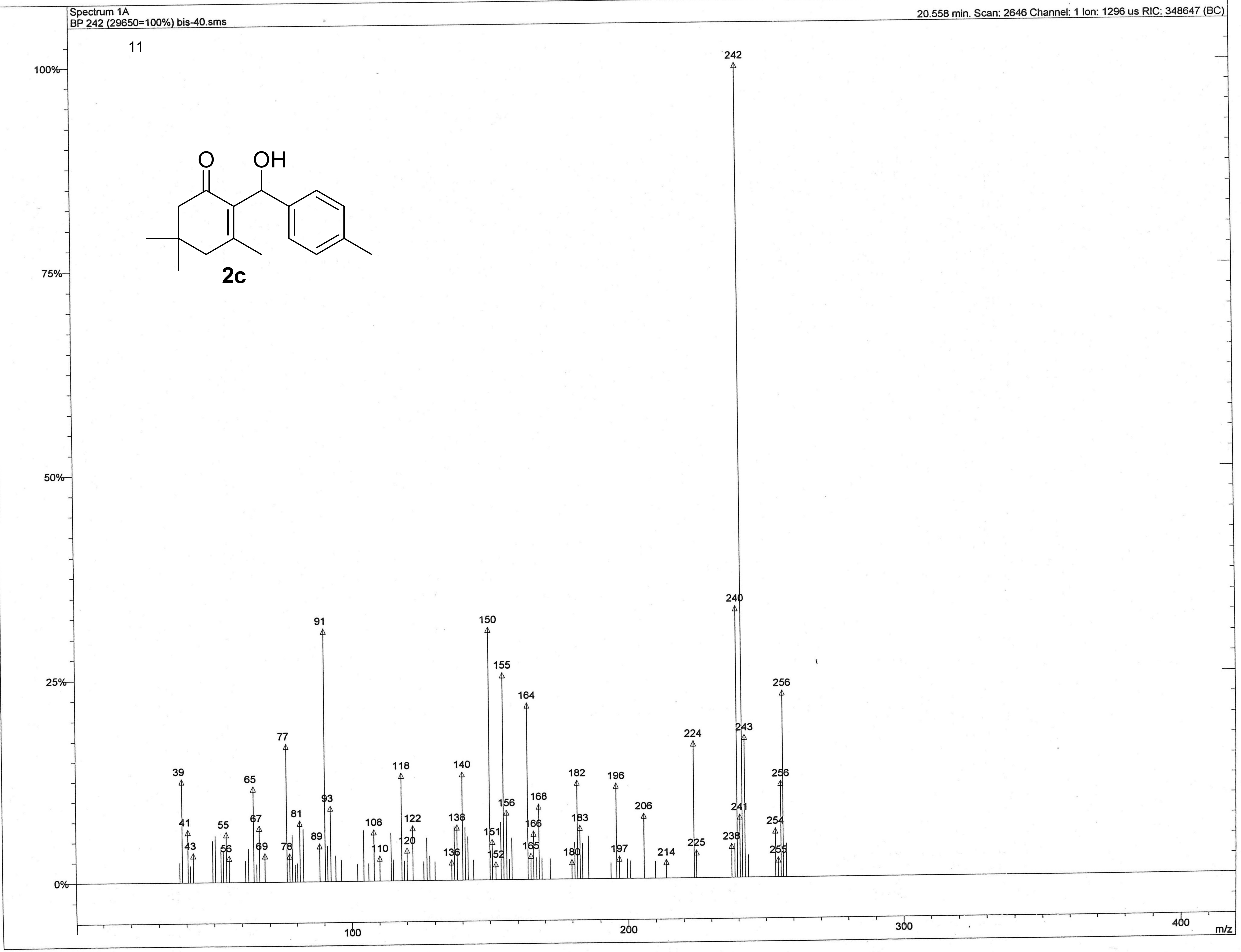
Spectrum 1A
BP 242 (29650=100%) bis-40.sms
20.558 min. Scan: 2646 Channel: 1 Ion: 1296 us RIC: 348647 (BC)
11
O
OH
2c
100%
75%
50%
25%
0%
242
240
91
150
155
164
256
243
224
77
39
65
118
140
182
196
256
93
156
168
206
241
41
55
67
81
108
122
138
166
183
254
89
151
120
69
56
43
78
110
136
152
165
180
197
214
225
238
255
100
200
300
400
m/z

12

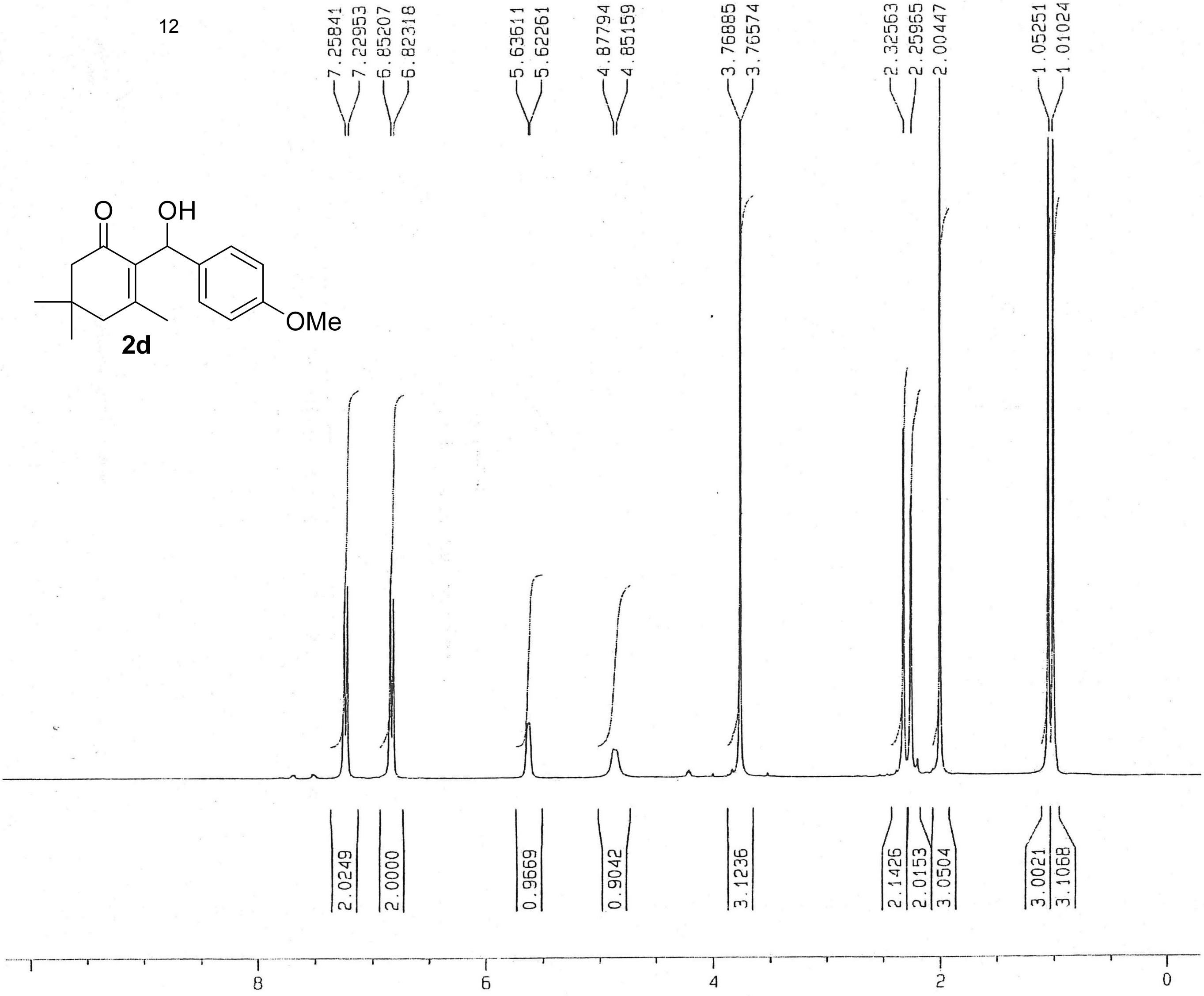
O
OH
OMe
2d
7.25841
7.22953
6.85207
6.82318
5.63611
5.62261
4.87794
4.85159
3.76885
3.76574
2.32563
2.25955
2.00447
1.05251
1.01024
2.0249
2.0000
0.9669
0.9042
3.1236
2.1426
2.0153
3.0504
3.0021
3.1068
8
6
4
2
0

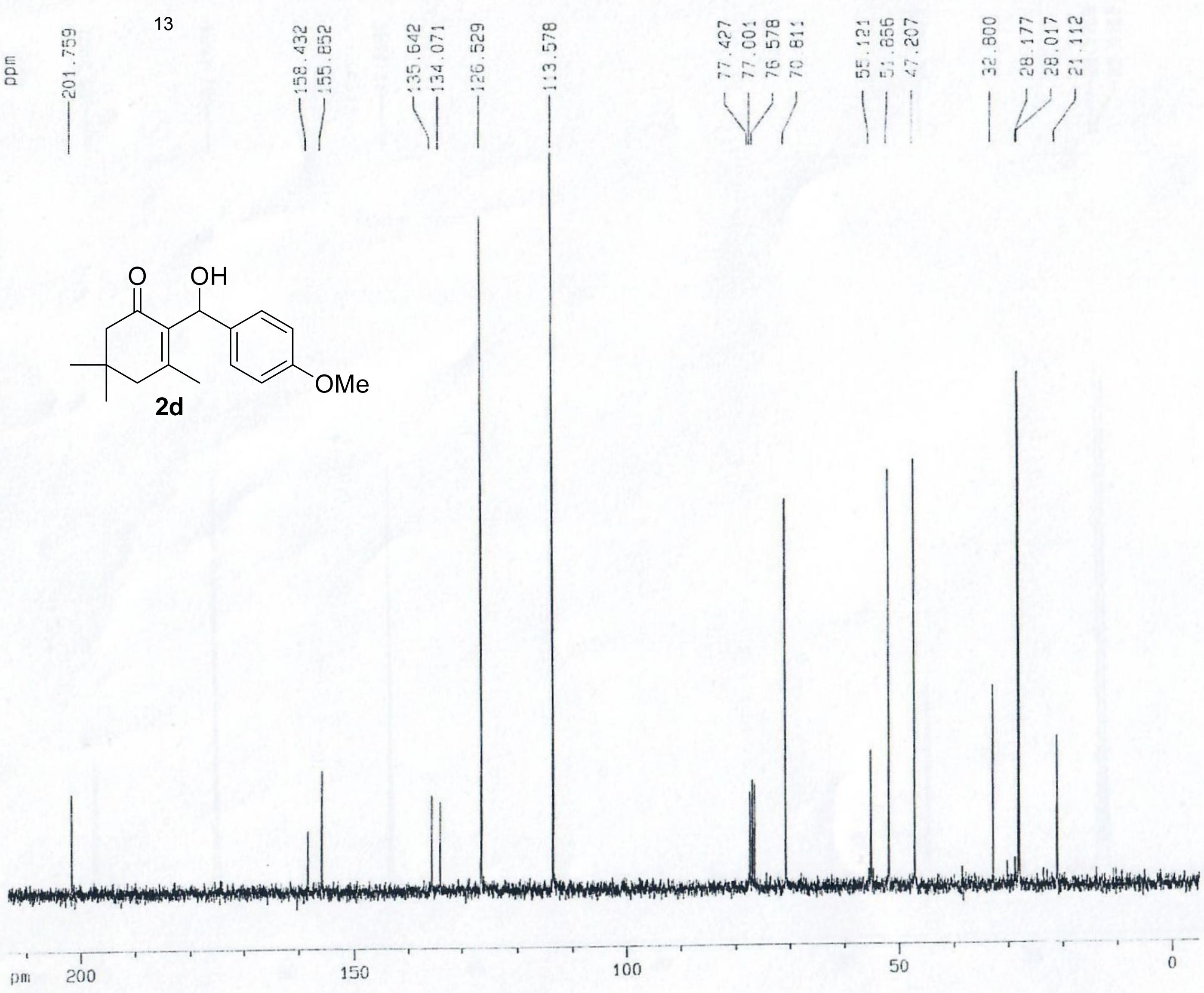
13
ppm
201.759
158.432
155.852
135.642
134.071
126.529
113.578
77.427
77.001
76.578
70.811
55.121
51.856
47.207
32.800
28.177
28.017
21.112
O
OH
OMe
2d
ppm 200
150
100
50
0

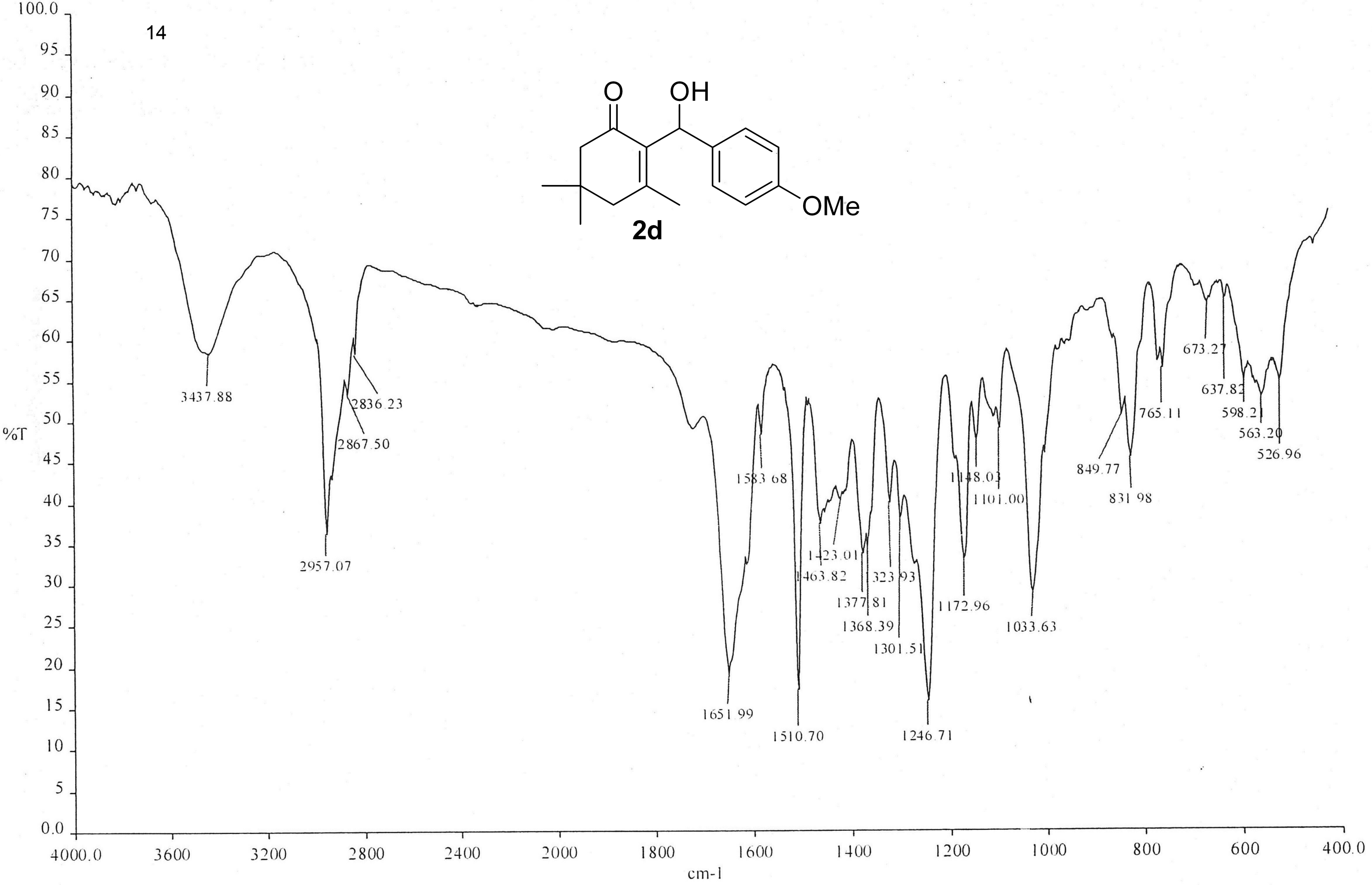

Ms. Akbarzadeh

Sample: bh(4-OMe)

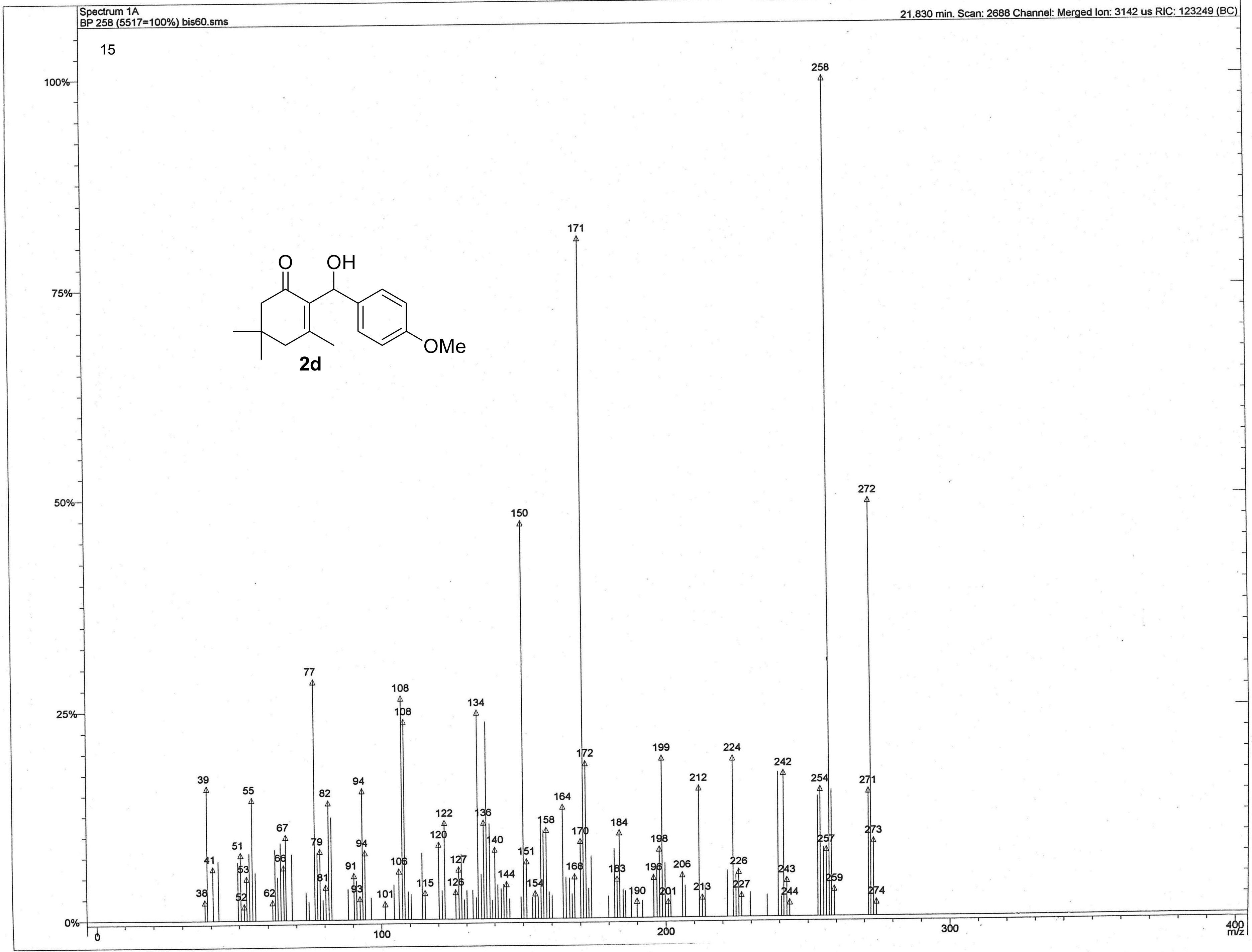
Spectrum 1A
BP 258 (5517=100%) bis60.sms
21.830 min. Scan: 2688 Channel: Merged Ion: 3142 us RIC: 123249 (BC)
15
100%
75%
50%
25%
0%
0
100
200
300
400
m/z
258
171
272
150
77
108
134
172
199
224
242
254
271
212
39
55
82
94
164
122
136
158
170
184
198
273
257
67
120
140
151
51
79
94
41
66
53
91
81
106
127
144
168
183
196
206
226
243
259
62
52
93
38
101
115
126
154
190
201
213
227
244
274
O
OH
OMe
2d

16

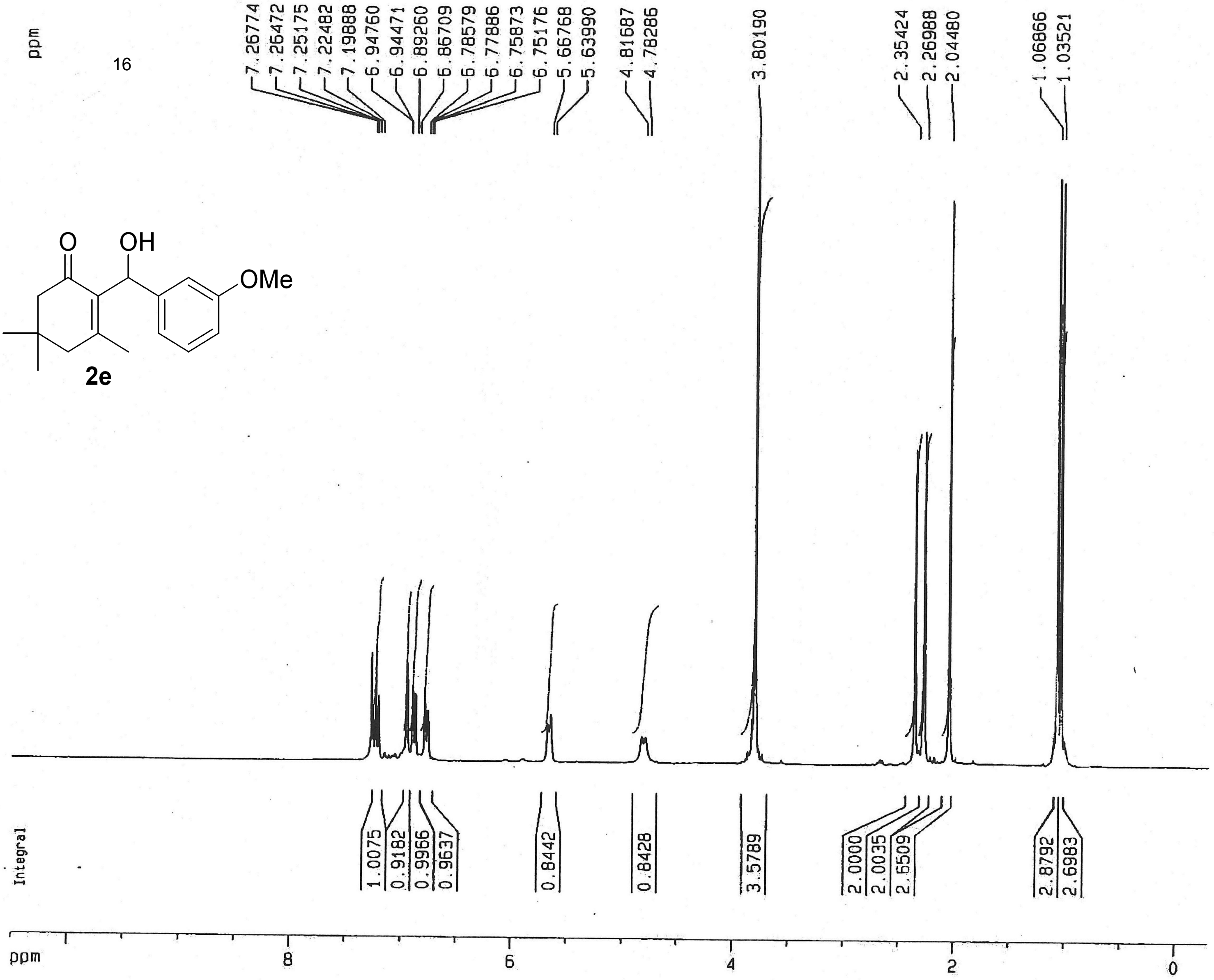
2e
ppm
7.26774
7.26472
7.25175
7.22482
7.19888
6.94760
6.94471
6.89260
6.86709
6.78579
6.77886
6.75873
6.75176
5.66768
5.63990
4.81687
4.78286
3.80190
2.35424
2.26988
2.04480
1.06866
1.03521
Integral
1.0075
0.9182
0.9966
0.9637
0.8442
0.8428
3.5789
2.0000
2.0035
2.6509
2.8792
2.6983
ppm
8
6
4
2
0

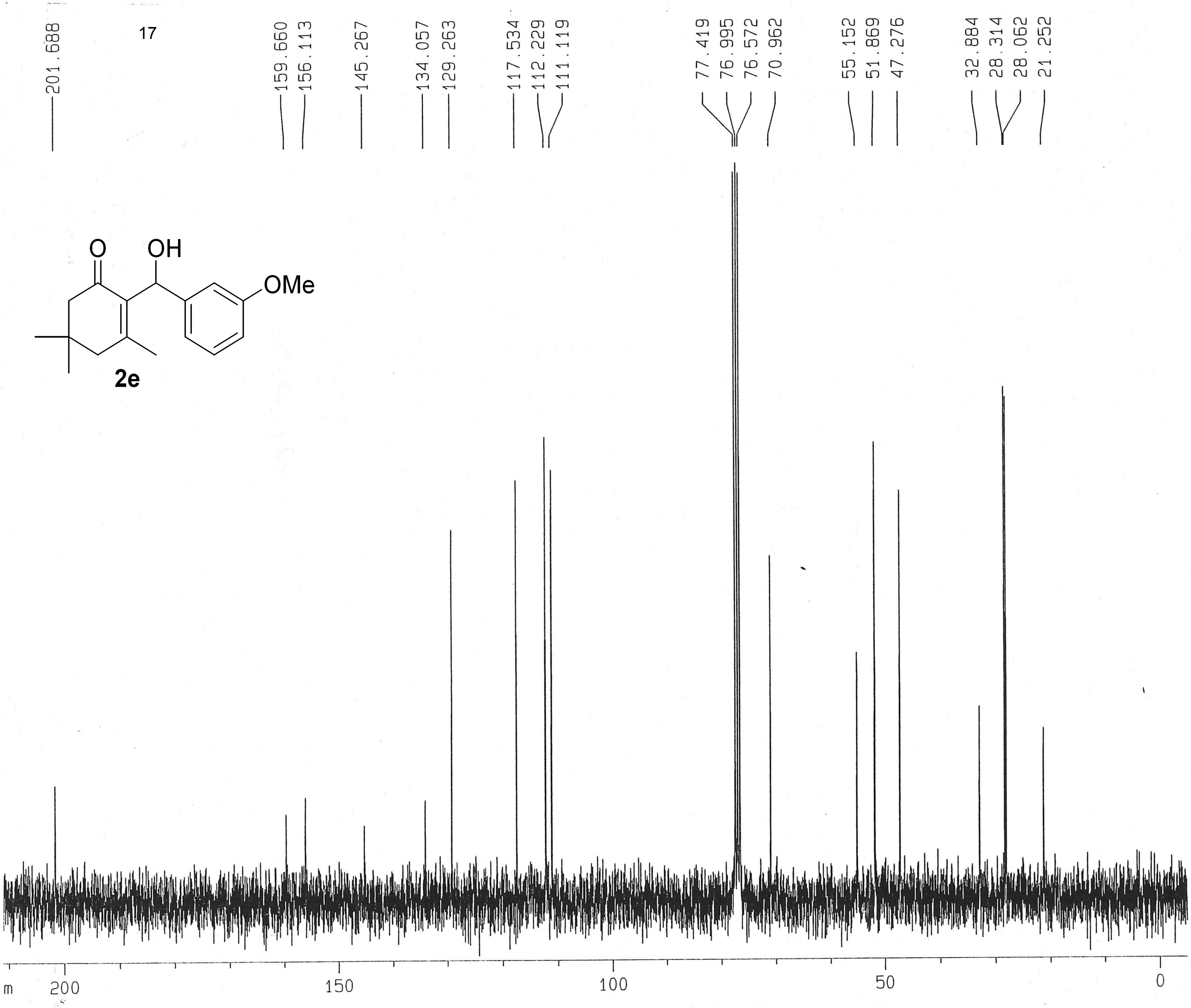
17
201.688
159.660
156.113
145.267
134.057
129.263
117.534
112.229
111.119
77.419
76.995
76.572
70.962
55.152
51.869
47.276
32.884
28.314
28.062
21.252
O
OH
OMe
2e
m
200
150
100
50
0

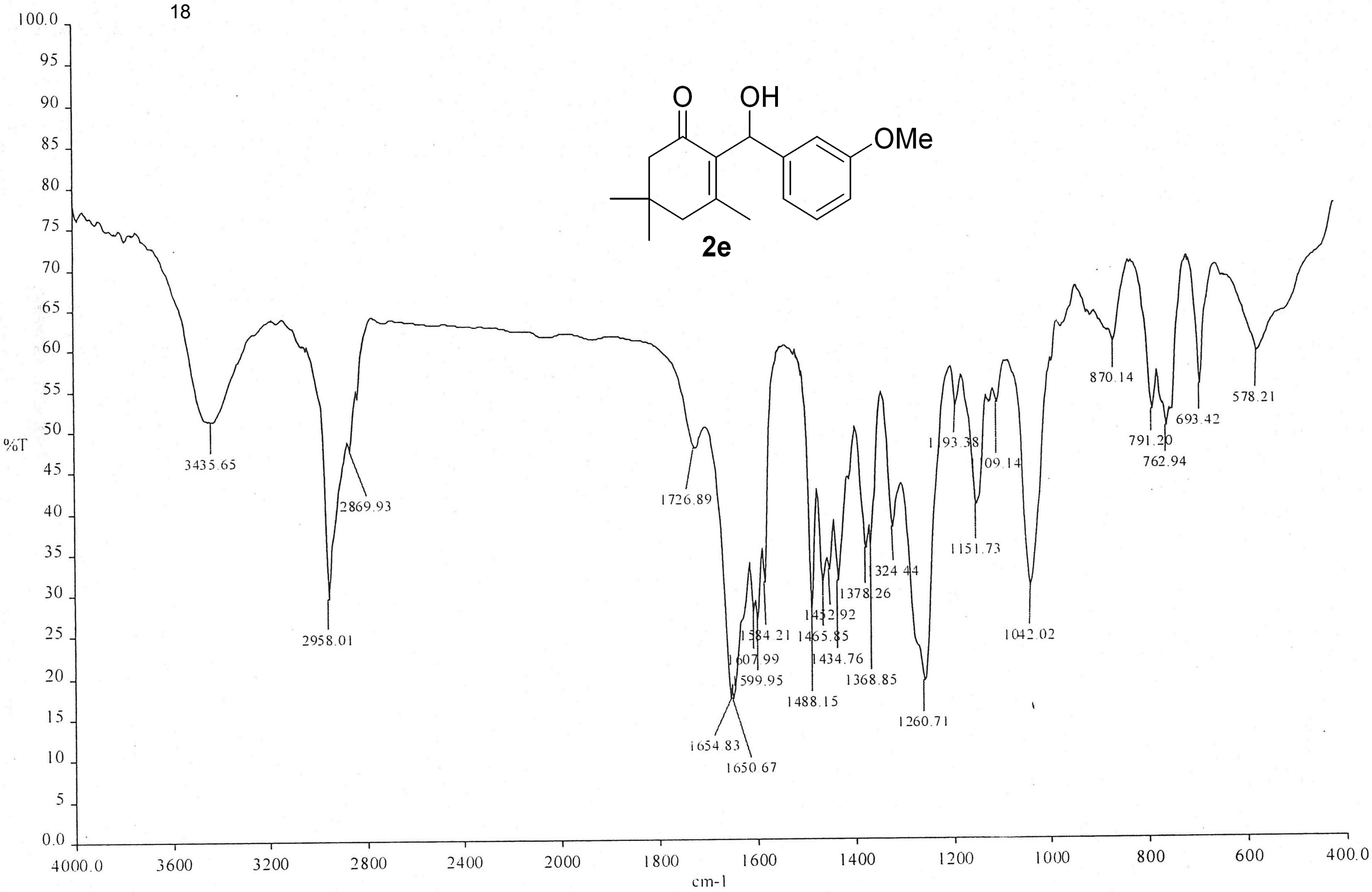

Ms. Akbarzade Sample: bis 36(bh)

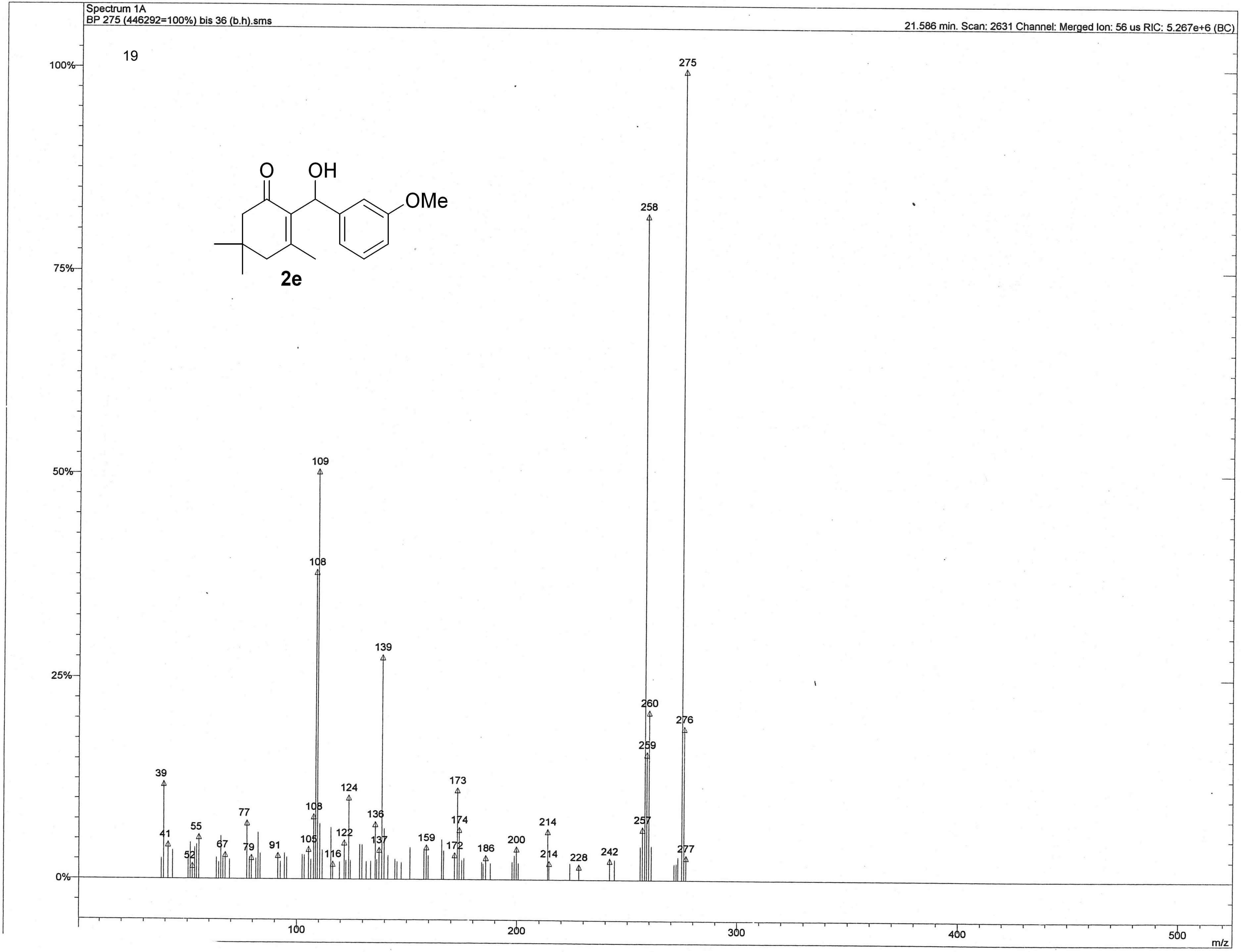
Spectrum 1A
BP 275 (446292=100%) bis 36 (b.h).sms
21.586 min. Scan: 2631 Channel: Merged Ion: 56 us RIC: 5.267e+6 (BC)
19
O
OH
OMe
2e
100%
75%
50%
25%
0%
275
258
109
108
139
260
276
259
39
41
52
55
67
77
79
91
105
108
116
122
124
136
137
159
172
173
174
186
200
214
214
228
242
257
277
100
200
300
400
500
m/z

2f

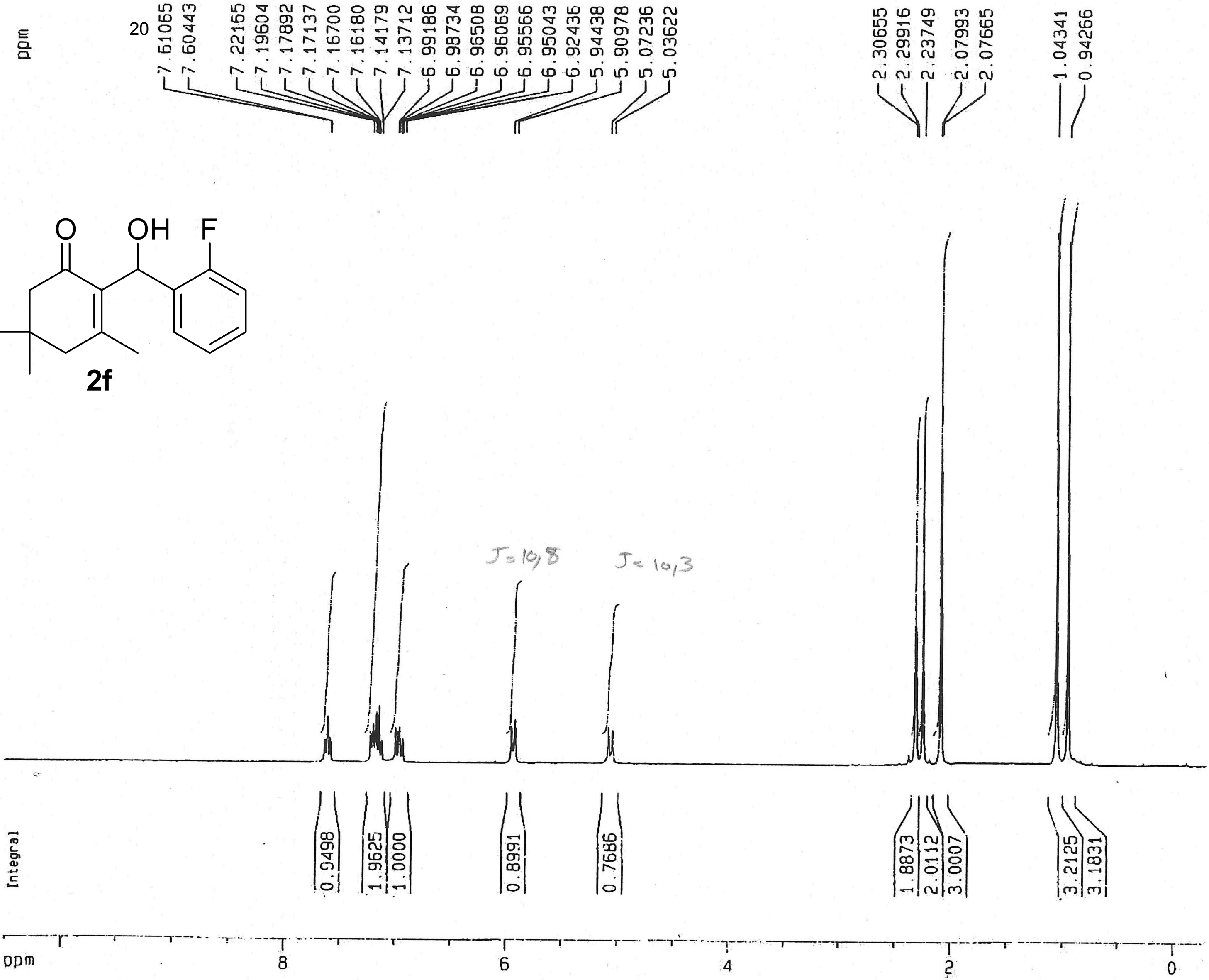
ppm
7.61065
7.60443
7.22165
7.19604
7.17892
7.17137
7.16700
7.16180
7.14179
7.13712
6.99186
6.98734
6.96508
6.96069
6.95566
6.95043
6.92436
5.94438
5.90978
5.07236
5.03622
2.30655
2.29916
2.23749
2.07993
2.07665
1.04341
0.94266
J=10,8
J=10,3
Integral
0.9498
1.9625
1.0000
0.8991
0.7686
1.8873
2.0112
3.0007
3.2125
3.1831
ppm
8
6
4
2
0

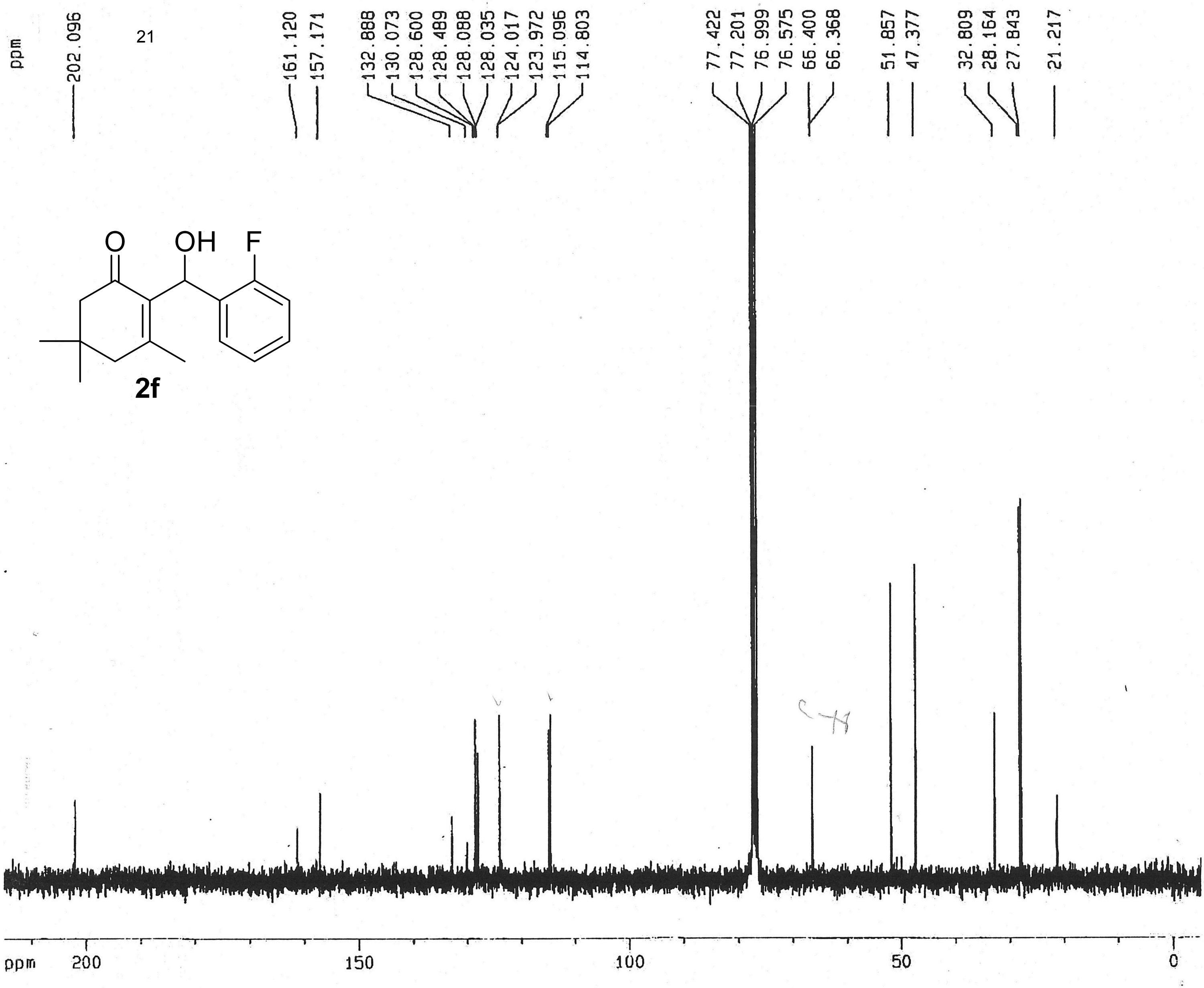

ppm
202.096
21
161.120
157.171
132.888
130.073
128.600
128.489
128.088
128.035
124.017
123.972
115.096
114.803
77.422
77.201
76.999
76.575
66.400
66.368
51.857
47.377
32.809
28.164
27.843
21.217
O
OH
F
2f
ppm
200
150
100
50
0

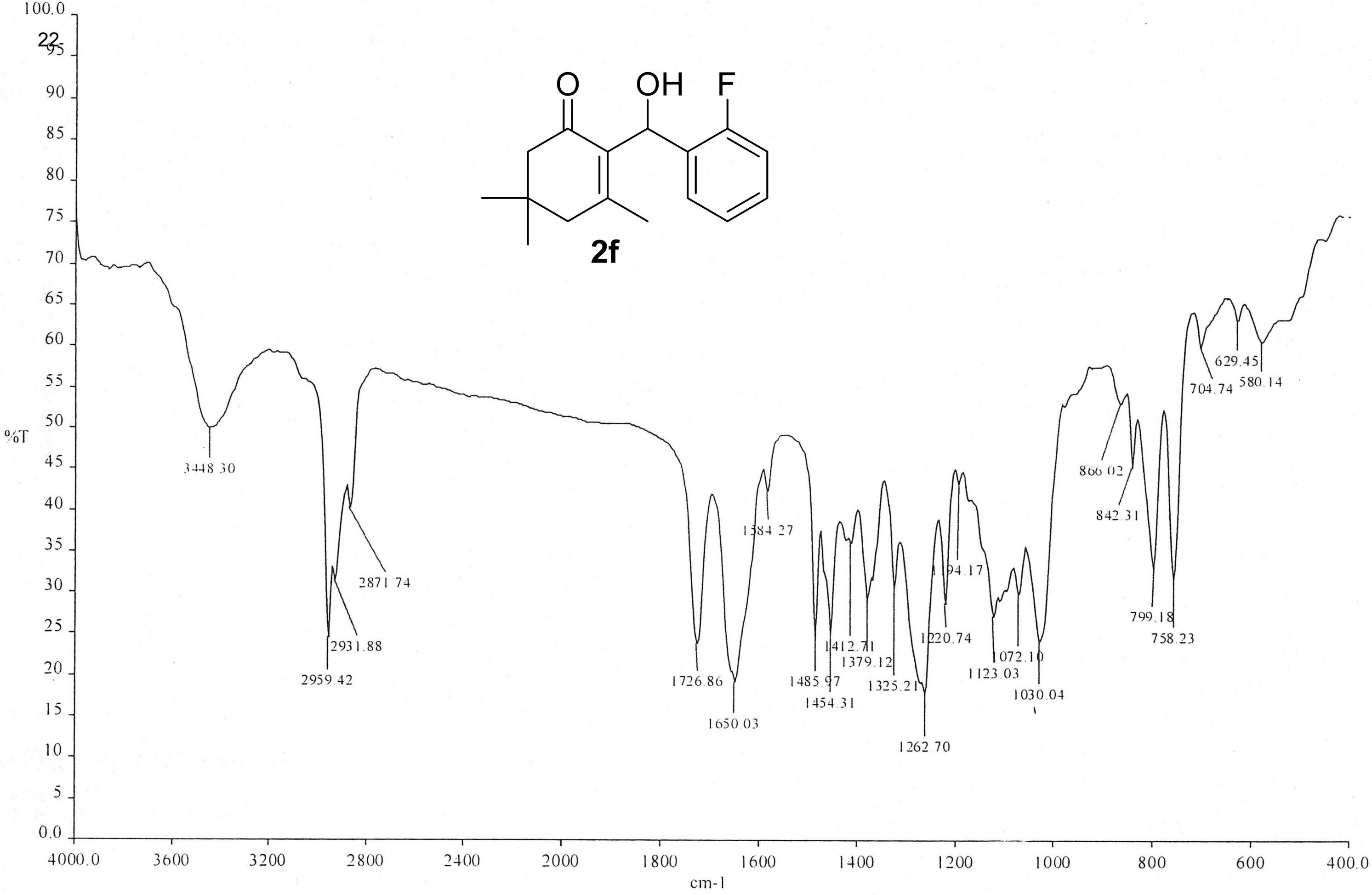

Ms. Akbarzade Sample: bhi11(up)

23

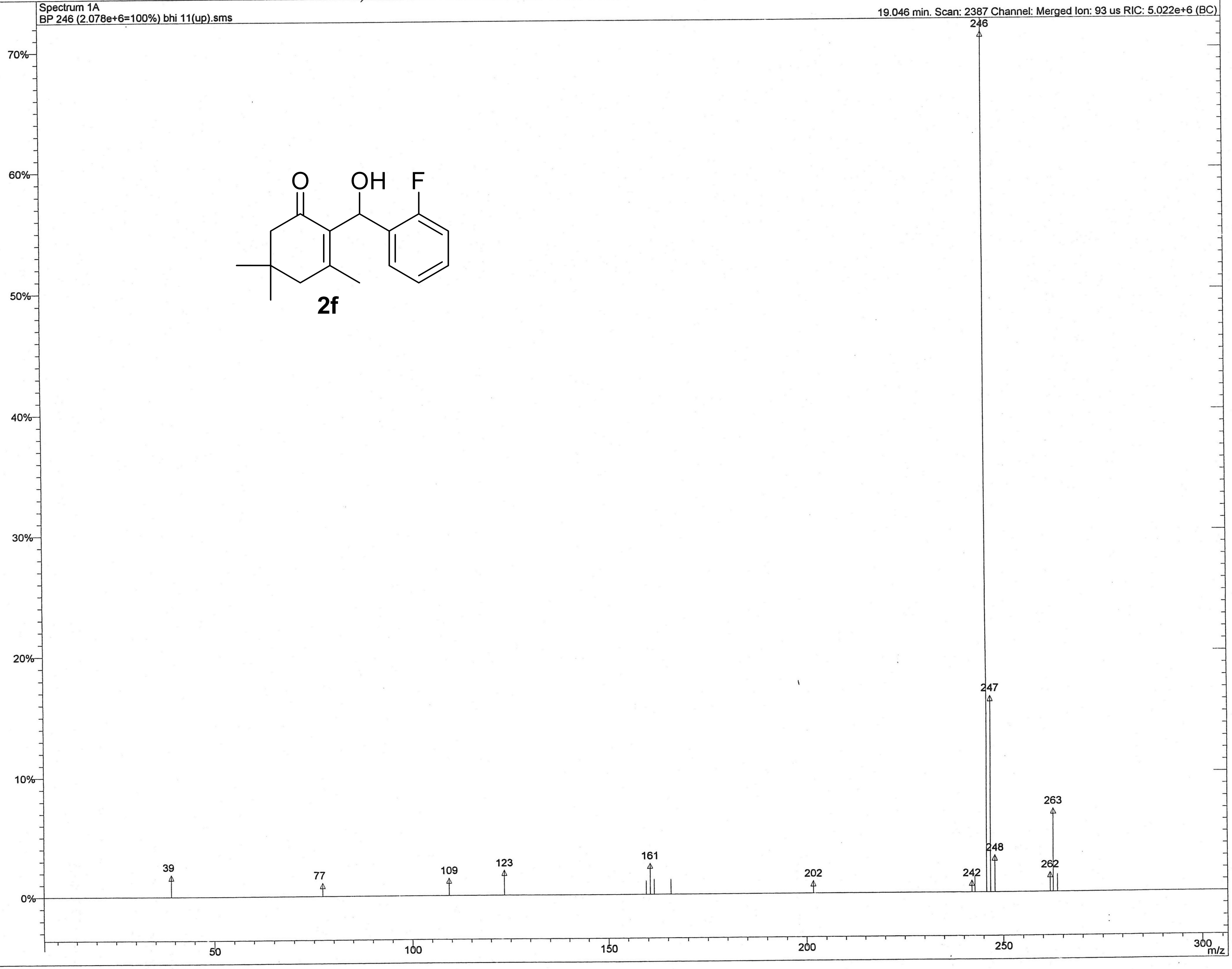
Spectrum 1A
BP 246 (2.078e+6=100%) bhi 11(up).sms
19.046 min. Scan: 2387 Channel: Merged Ion: 93 us RIC: 5.022e+6 (BC)
70%
60%
50%
40%
30%
20%
10%
0%
O
OH
F
2f
246
247
263
248
262
242
202
161
123
109
77
39
50
100
150
200
250
300
m/z

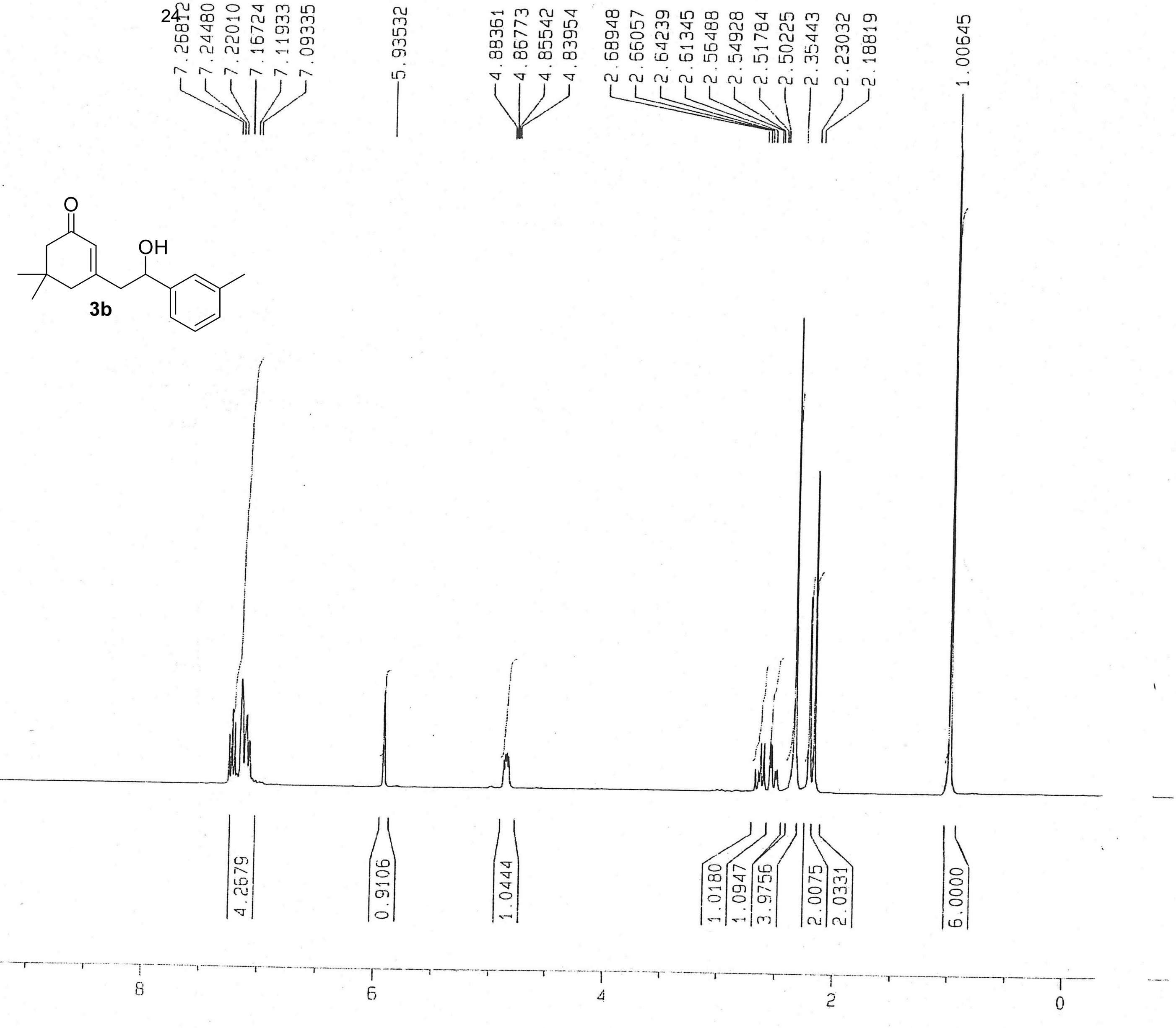
24
7.26812
7.24480
7.22010
7.16724
7.11933
7.09335
5.93532
4.88361
4.86773
4.85542
4.83954
2.68948
2.66057
2.64239
2.61345
2.55488
2.54928
2.51784
2.50225
2.35443
2.23032
2.18819
1.00645
O
OH
3b
4.2579
0.9106
1.0444
1.0180
1.0947
3.9756
2.0075
2.0331
6.0000
8
6
4
2
0

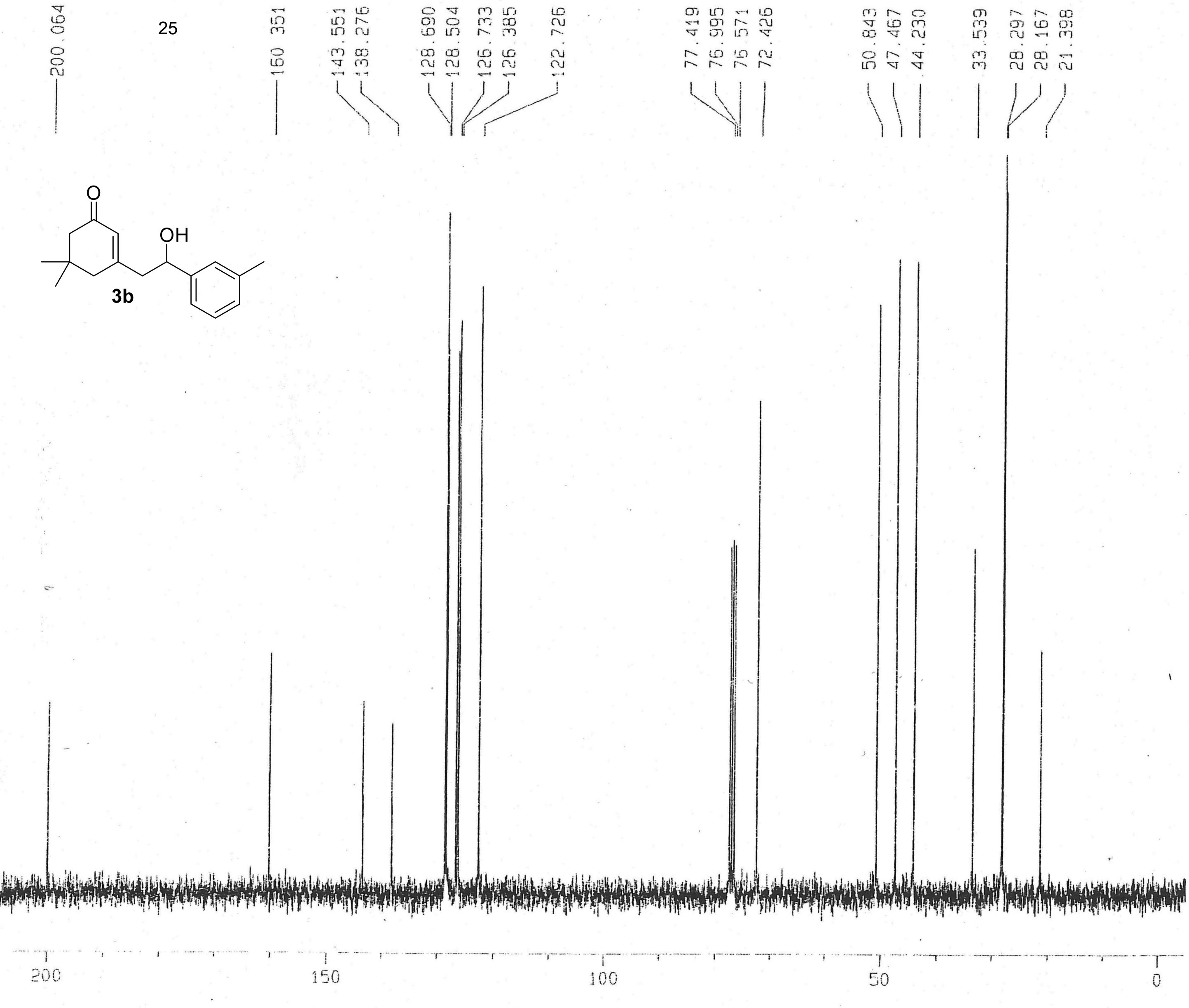
200.064
160.351
143.551
138.276
128.690
128.504
126.733
126.385
122.726
77.419
76.995
76.571
72.426
50.843
47.467
44.230
33.539
28.297
28.167
21.398
O
OH
3b
200
150
100
50
0

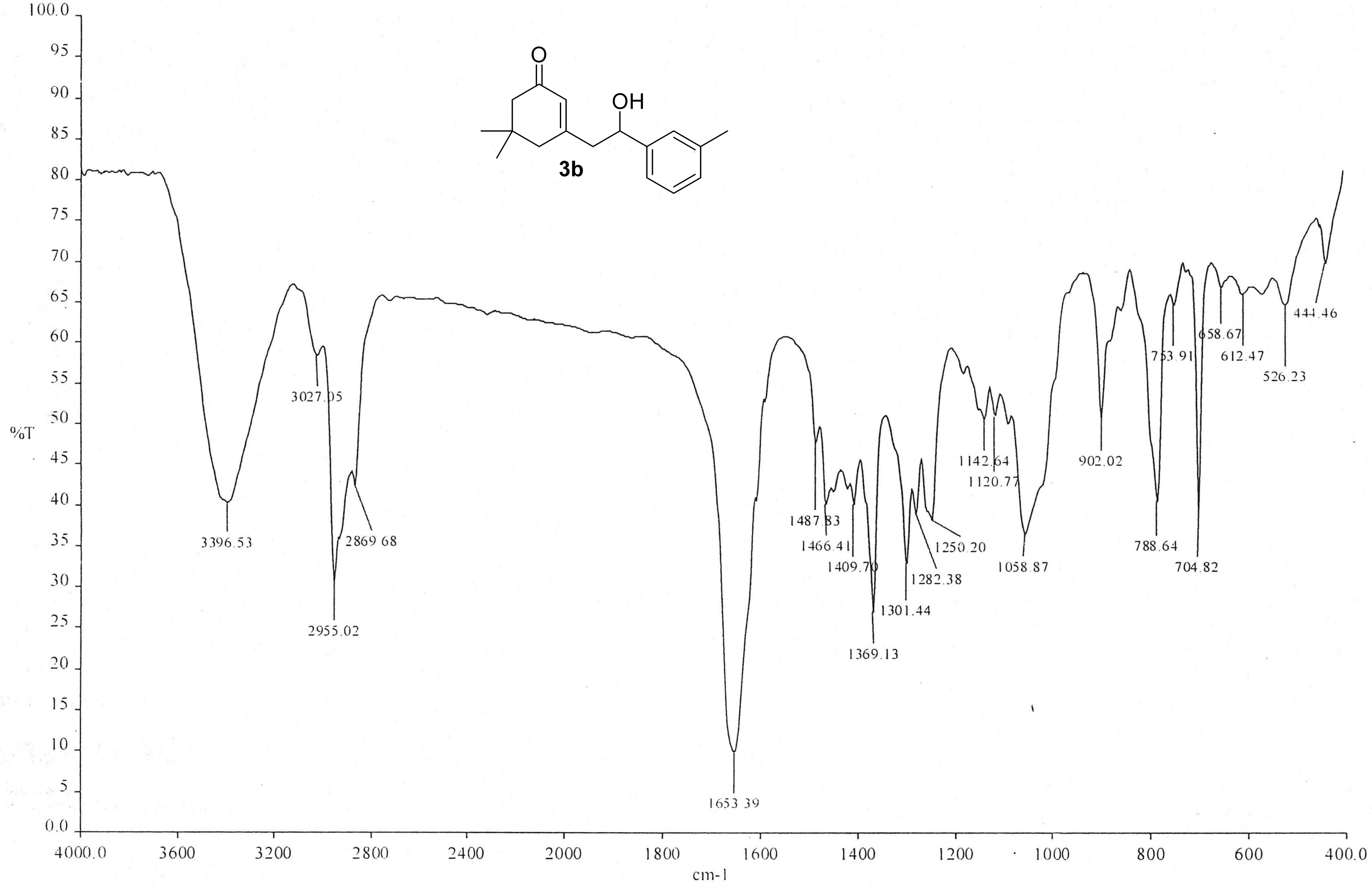

Ms. Akbarzade Sample: bis 34 aldol

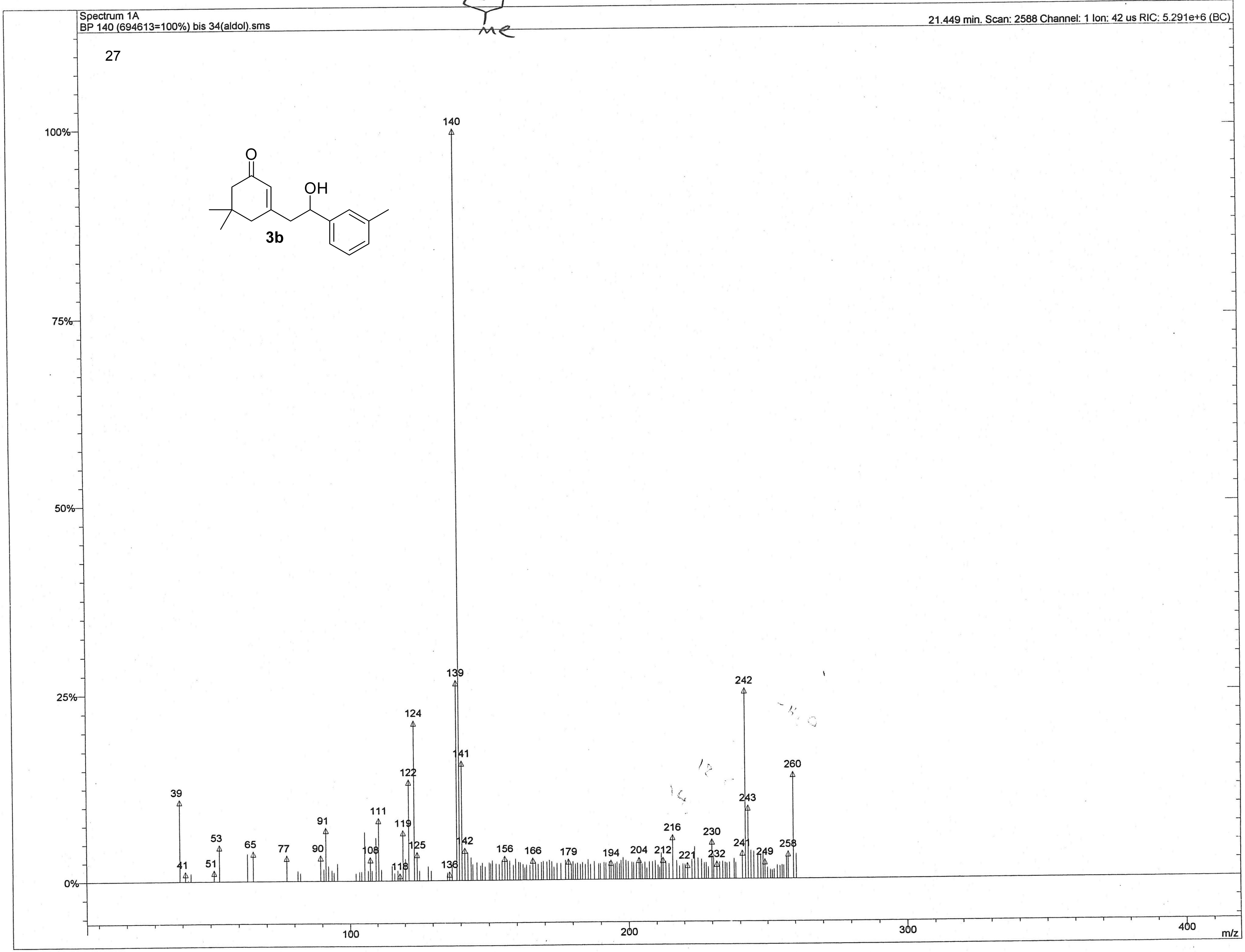
Spectrum 1A
BP 140 (694613=100%) bis 34(aldol).sms
21.449 min. Scan: 2588 Channel: 1 Ion: 42 us RIC: 5.291e+6 (BC)
27
O
OH
3b
100%
75%
50%
25%
0%
140
139
124
122
141
242
260
243
39
111
119
91
216
230
53
65
77
90
108
125
142
156
166
179
194
204
212
221
232
241
249
258
41
51
118
136
100
200
300
400
m/z

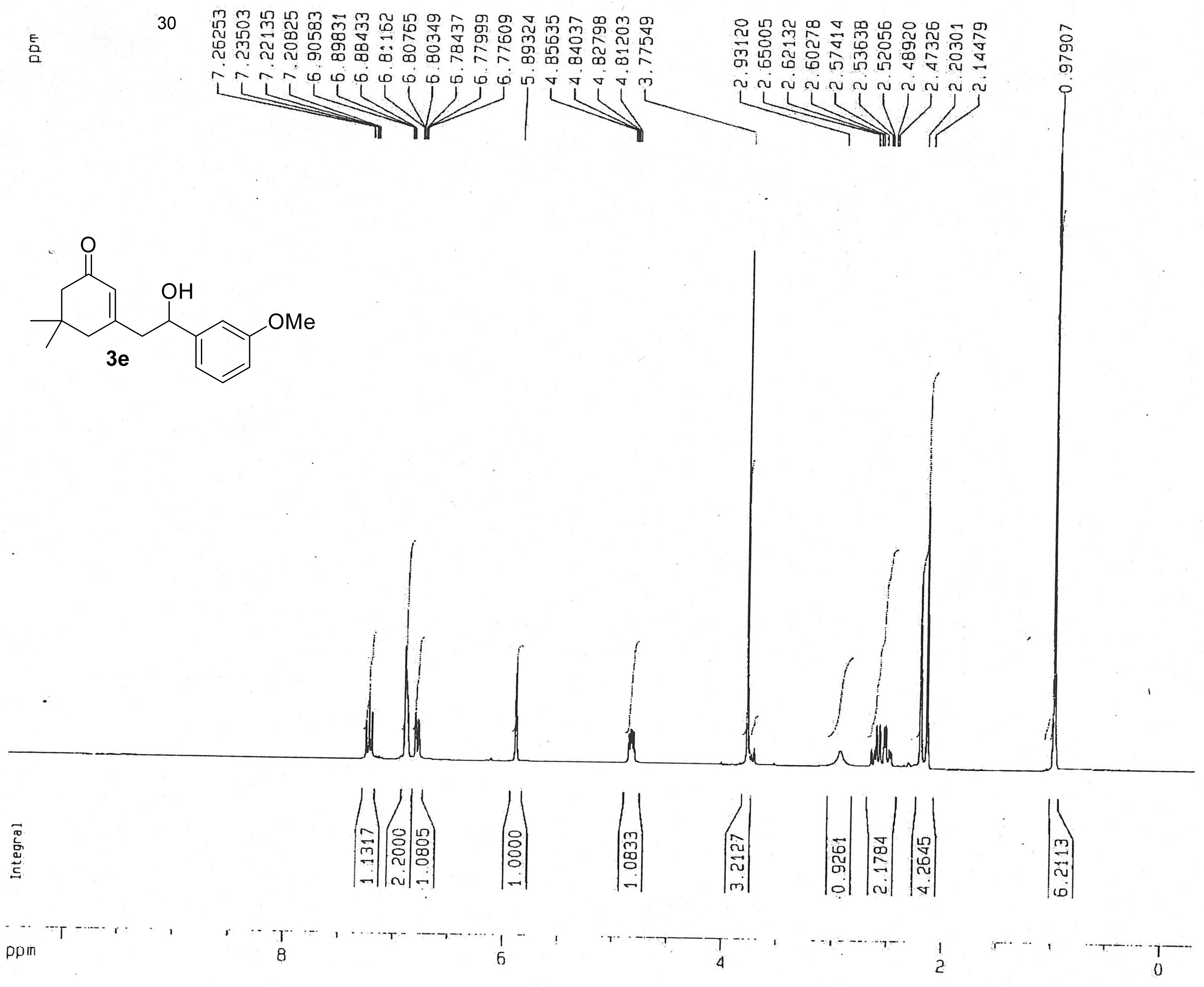
30
ppm
7.26253
7.23503
7.22135
7.20825
6.90583
6.89831
6.88433
6.81162
6.80765
6.80349
6.78437
6.77999
6.77609
5.89324
4.85635
4.84037
4.82798
4.81203
3.77549
2.93120
2.65005
2.62132
2.60278
2.57414
2.53638
2.52056
2.48920
2.47326
2.20301
2.14479
0.97907
O
OH
OMe
3e
Integral
1.1317
2.2000
1.0805
1.0000
1.0833
3.2127
0.9261
2.1784
4.2645
6.2113
ppm
8
6
4
2
0

31

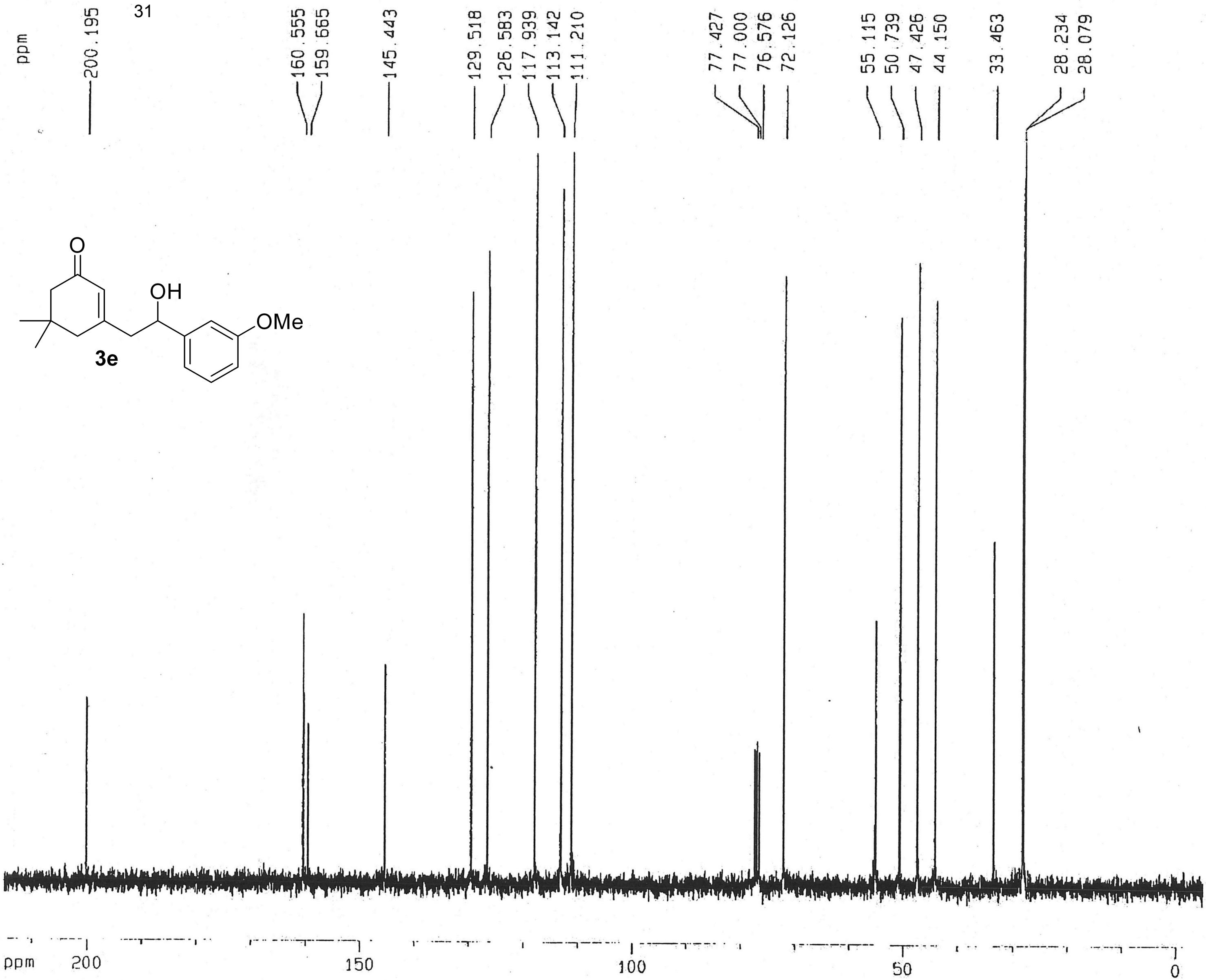
ppm
200.195
160.555
159.665
145.443
129.518
126.583
117.939
113.142
111.210
77.427
77.000
76.576
72.126
55.115
50.739
47.426
44.150
33.463
28.234
28.079
O
OH
OMe
3e
ppm
200
150
100
50
0

32

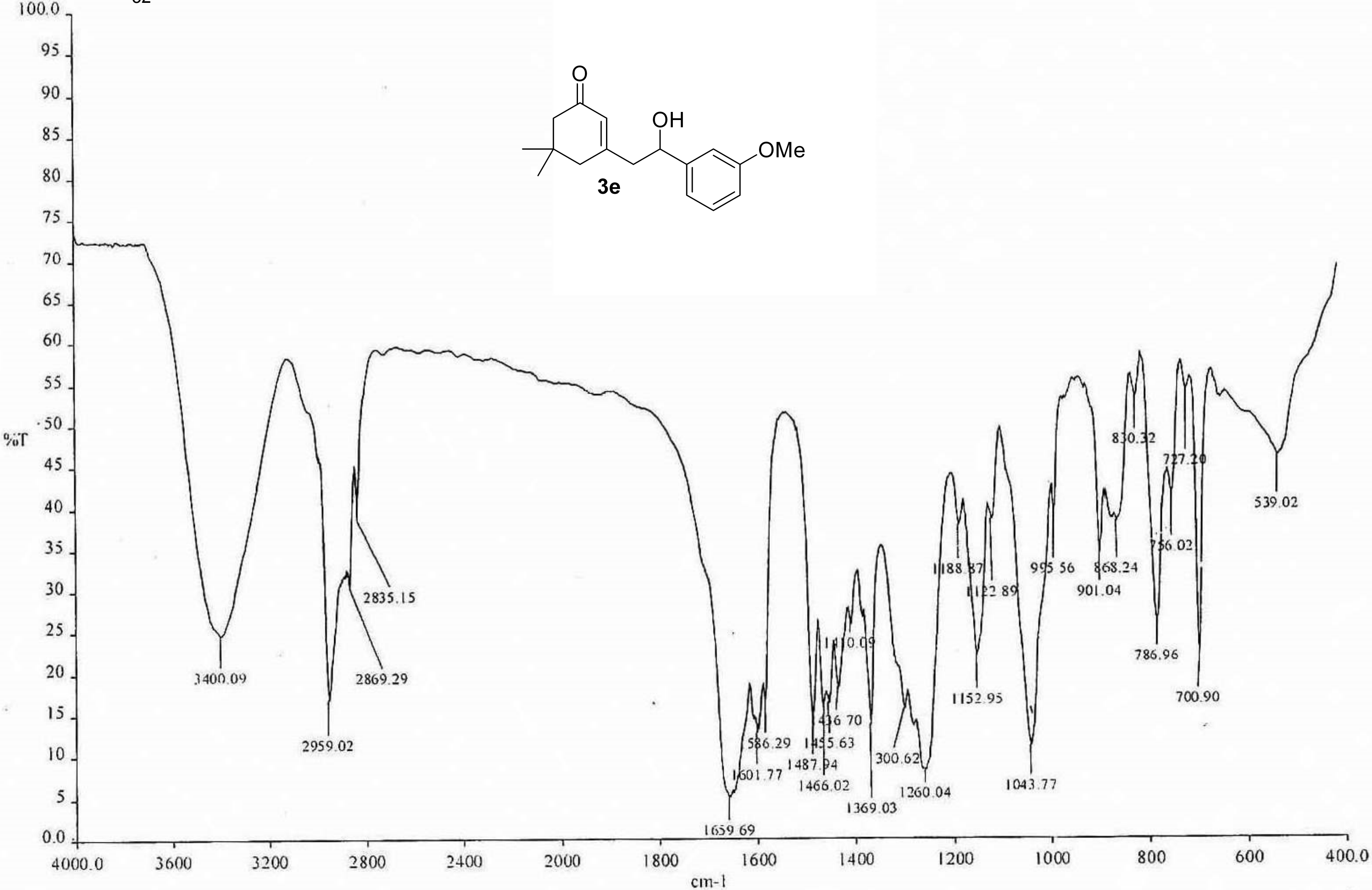

O
OH
OMe
3e
%T
cm-1
3400.09
2959.02
2869.29
2835.15
1659.69
1601.77
1487.94
1466.02
1369.03
1260.04
1152.95
1188.87
1043.77
995.56
901.04
868.24
830.32
786.96
756.02
727.20
700.90
539.02

Spectrum Plot - 2/4/2013 11:27 AM

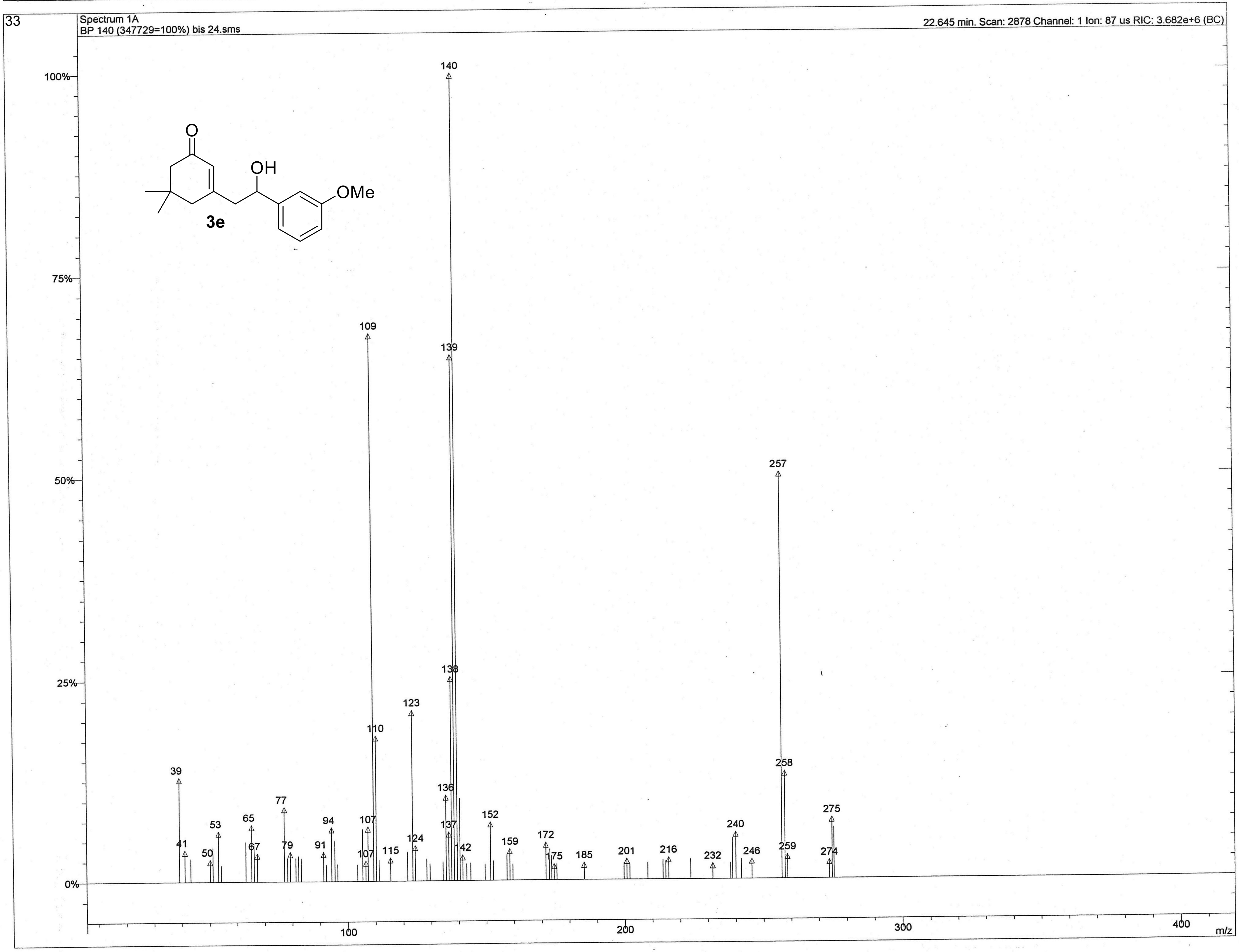

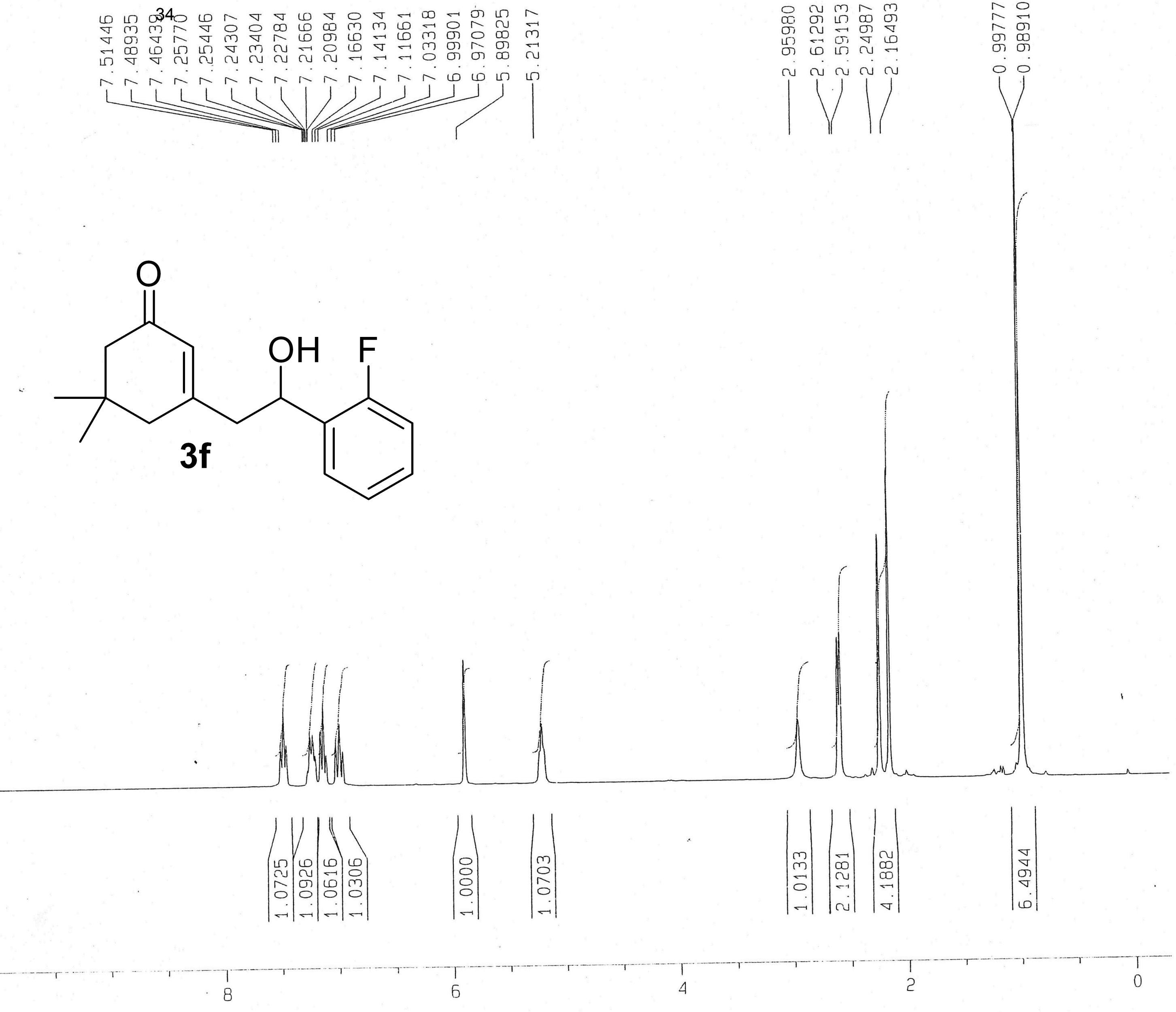
34
7.51446
7.48935
7.46439
7.25770
7.25446
7.24307
7.23404
7.22784
7.21666
7.20984
7.16630
7.14134
7.11661
7.03318
6.99901
6.97079
5.89825
5.21317
2.95980
2.61292
2.59153
2.24987
2.16493
0.99777
0.98910
O
OH
F
3f
1.0725
1.0926
1.0616
1.0306
1.0000
1.0703
1.0133
2.1281
4.1882
6.4944
8
6
4
2
0

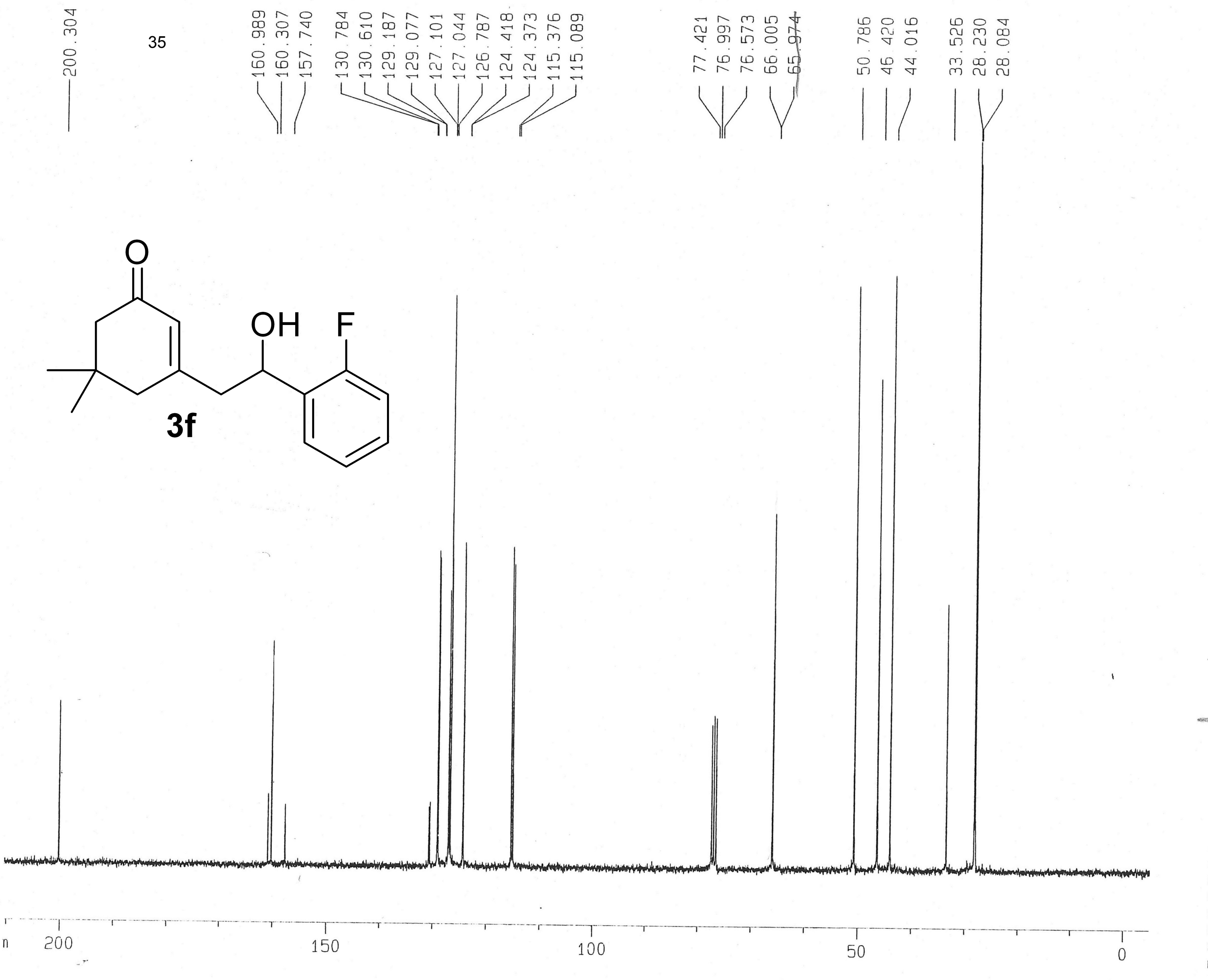

200.304
160.989
160.307
157.740
130.784
130.610
129.187
129.077
127.101
127.044
126.787
124.418
124.373
115.376
115.089
77.421
76.997
76.573
66.005
65.974
50.786
46.420
44.016
33.526
28.230
28.084
O
OH
F
3f
200
150
100
50
0

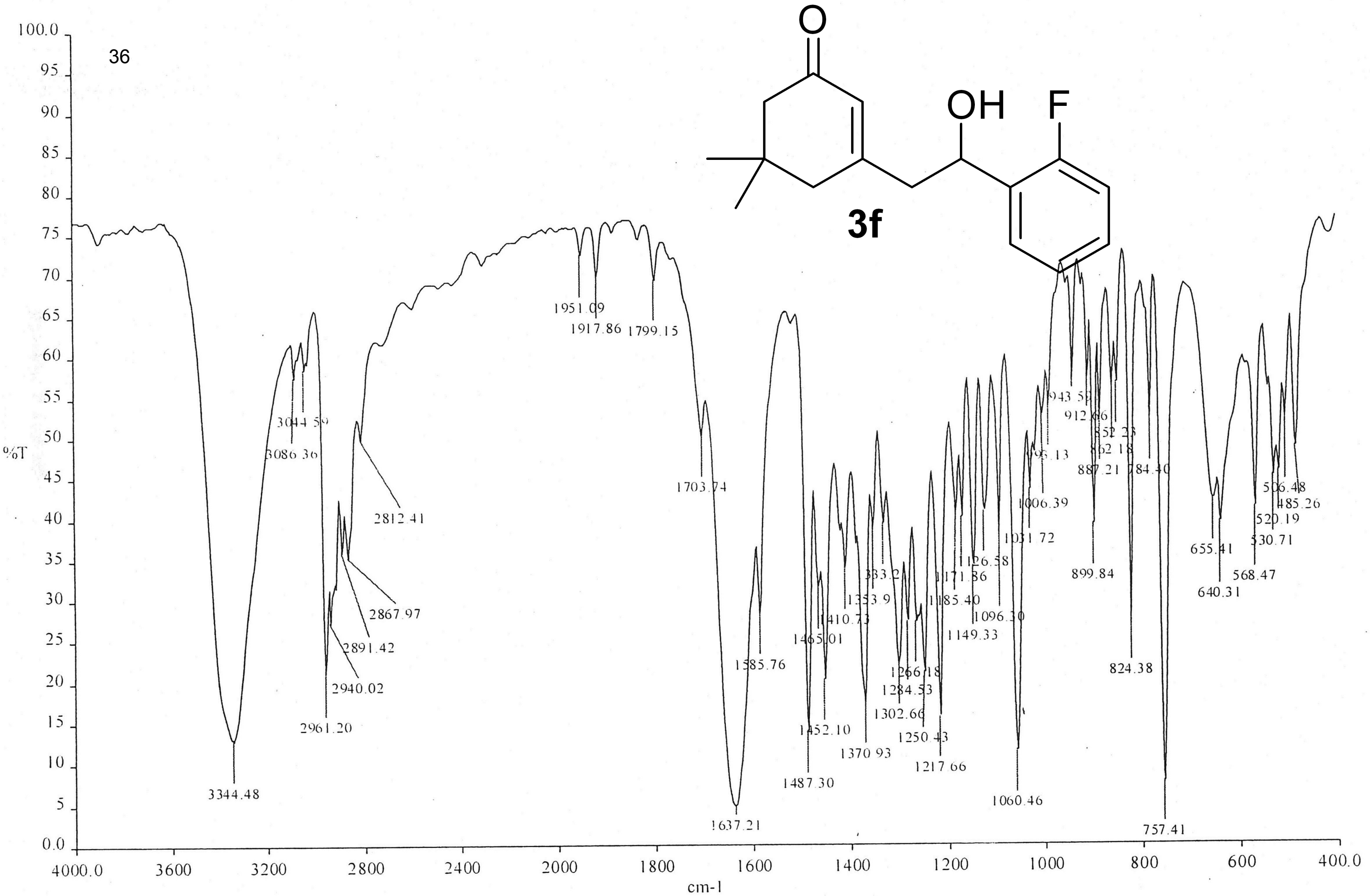

Ms. Akbarzade Sample: bhi(11)down

Print Date: 02 Jan 2013 13:51:14
37

Spectrum Plot - 1/2/2013 1:51 PM

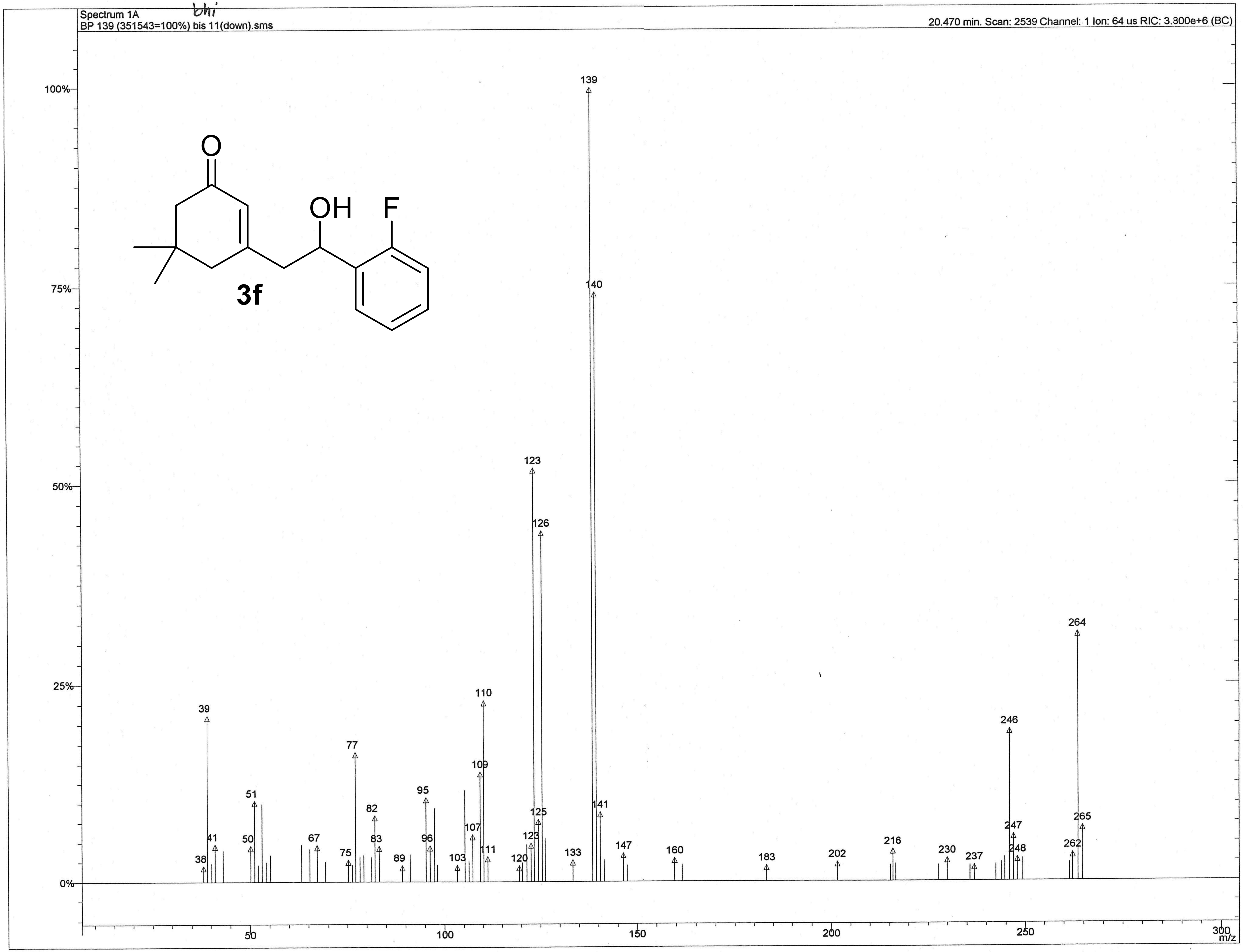

Supplement: Supplementary file 1 — Supporting Information [file OPEN-14-e202500040-s001.pdf]
